# Minimizing Batch Effects in Mass Cytometry Data

**DOI:** 10.3389/fimmu.2019.02367

**Published:** 2019-10-15

**Authors:** Ronald P. Schuyler, Conner Jackson, Josselyn E. Garcia-Perez, Ryan M. Baxter, Sidney Ogolla, Rosemary Rochford, Debashis Ghosh, Pratyaydipta Rudra, Elena W. Y. Hsieh

**Affiliations:** ^1^Department of Immunology and Microbiology, University of Colorado School of Medicine, Aurora, CO, United States; ^2^Department of Biostatistics and Informatics, Colorado School of Public Health, Aurora, CO, United States; ^3^Department of Statistics, Oklahoma State University, Stillwater, OK, United States; ^4^Division of Allergy and Immunology, Department of Pediatrics, University of Colorado School of Medicine, Children's Hospital Colorado, Aurora, CO, United States

**Keywords:** normalization, barcode, anchor, mass cytometry, clinical studies, human immunology

## Abstract

Cytometry by Time-Of-Flight (CyTOF) uses antibodies conjugated to isotopically pure metals to identify and quantify a large number of cellular features with single-cell resolution. A barcoding approach allows for 20 unique samples to be pooled and processed together in one tube, reducing the intra-barcode technical variability. However, with only 20 samples per barcode, multiple barcode sets (batches) are required to address questions in robustly powered study designs. A batch adjustment procedure is required to reduce variability across batches and to facilitate direct comparison of runs performed across multiple barcodes run over weeks, months, or years. We describe a method using technical replicates that are included in each run to determine and apply an appropriate adjustment per batch without manual intervention. The use of technical replicate samples (i.e., anchors or reference samples) avoids assumptions of sample homogeneity among batches, and allows direct estimation of batch effects and appropriate adjustment parameters applicable to all samples within a batch. Quantification of cell subpopulations and mean signal intensity pre- and post-adjustment using both manual gating and unsupervised clustering demonstrate substantial mitigation of batch effects in the anchor samples used for this adjustment calculation, and in a second validation set of technical replicates.

## Introduction

Mass cytometry, or Cytometry by Time-Of-Flight (CyTOF), is a high-throughput single-cell analysis technology that allows simultaneous measurement of 40+ cellular parameters via detection of rare earth heavy metal isotopes conjugated to monoclonal antibodies. The high-dimensionality of mass cytometry casts a wide exploratory net to allow: (1) discovery of novel cell phenotypes, (2) quantification of differences in cell-type composition (population frequency), and (3) analysis of marker expression levels (mean signal intensity) that may reflect different activation and/or functional cellular attributes. This systems immunology approach, when applied with relevant computational strategies, has the potential to unravel the cellular diversity and heterogeneity that underlies human immune-mediated disorders, providing descriptive and mechanistic insight with translational impact. For example, mass cytometry has been used to study human B cell development in the context of healthy and abnormal progression in acute lymphoblastic leukemia, leading to improved predictive risk stratification methods (1, 2). Mass cytometry applications to understand dysregulated signaling networks and downstream cytokine production in pediatric SLE have demonstrated unique signatures that may help with diagnosis and monitoring response to therapy (3, 4). Additionally, mass cytometry has been applied to study T cell proliferation and differentiation (5), macrophage phagocytosis (6), parallel DNA, RNA, and protein biosynthesis (7), and cell cycle status in malignancy and immunotherapy (8–10), to name a few applications.

The ability of mass cytometry to address a wide variety of biological questions related to immune cellular phenotype and function illustrates a desire for the technology to be utilized in increasingly complex study designs, with increasing number of study subjects, *in vivo* therapeutic interventions, *in vitro* conditions, and different disease status/timepoints. Differences in immune cell subset abundance or functionality can be explored among multiple patient groups of different diagnosis, between patient groups receiving different therapeutic interventions, or the same interventions across therapy phases. Addressing such questions often requires multiple patients per group, and often multiple samples (accounting for *in vitro* stimulation conditions and/or incubation timepoints) per patient. Additionally, given that mass cytometry measures hundreds of cells per second, and the ideal goal data collection numbers range in the hundreds of thousands of cells per sample (or more if analyzing rare cell types), instrument run times would expand over days or weeks for well-powered studies. Inherent to human immunology studies, prospective sample collection often occurs over the course of months to years. Therefore, the combination of the need of multiple samples to be analyzed per project, the relative speed of data acquisition on the instrument, and the prospective nature of the sample collection in human studies, requires the ability to process and run samples in multiple batches. A barcoding approach allows for multiple samples to be stained together in one tube, reducing the intra-barcode technical variability, and optimizing data acquisition speed and efficiency (decreased cell loss) as it constitutes a single sample run on the instrument (11). However, at 20 samples per barcode set, multiple barcode sets (batches) are still required to address questions in robustly powered study designs. While a barcoding approach does reduce technical variability among the 20 samples within the barcode set, having multiple barcode sets adds inter-barcode variability. Variability in reagent lots, instrument maintenance and calibration (detector changes, instrument repairs), antibody concentration to cell number ratio per barcode set, and other technical issues related to sample preparation can introduce artifacts and complicate analysis of samples processed and run in different barcode sets. The antibody reagent variability issue can be potentially overcome by using lyophilized antibody cocktails (12, 13), or preparing master mixes of antibody panels that are aliquoted, frozen, and later distributed across barcode sets over time (14). Bead normalization addresses signal variation issues related to instrument changes (15), however it does not address the other factors related to batch-to-batch variability mentioned above.

To facilitate analysis of large-scale mass cytometry experiments and allow for data analysis across prospective longitudinal studies, a batch adjustment process is required to reduce variability among batches/barcode sets. Batch adjustment will allow for direct comparison of data acquired on barcode sets across time. Batch effects are ubiquitous in high-throughput experiments (e.g., RNA sequencing, proteomics, metabolomics), and methods to adjust for these effects have been developed for domain-specific application, as well as general purpose frameworks (16). Surrogate variable analysis (SVA) identifies latent factors in a data set using the singular value decomposition of a matrix. Latent factors represent coordinated modules within the data and may correspond to biological effects, batch effects, or other sources of systematic variability (16). ComBat uses a Bayesian framework to model data, including covariates for batch, and possibly latent variables identified by SVA (17, 18). These effects are then removed using regression. Remove unwanted variation (RUV) uses spike-in control samples or genes which are expected to remain relatively constant between batches to estimate technical effects (19). Combat, Surrogate Variable Analysis (SVA) and related methodologies for batch effect adjustment cannot be directly applied to mass cytometry data for the following reasons. First, they require a data matrix in which the rows correspond to genes or molecules, while the columns are samples. A single mass cytometry sample is inherently more complex, as it consists of many single cell events, each of which is represented as a vector of measurements (ion counts from each mass channel). The inherent structure of multiple batches, with multiple samples, each with many single-cell events is not naturally represented in the two-dimensional matrix format that SVA and Combat assume. It is possible to consider each single-cell event as a sample and simply concatenate all events from all samples into a single matrix, but with hundreds of thousands of cells per sample, many samples per batch, and many batches, this approach does not scale well with increasing experiment size. Second, it is important to understand the proper data distribution of mass cytometry experiments before applying batch adjustment methods. This is still an open question and needs further investigation. Higher-dimensional tensor decomposition methods have been developed (20) and could be adapted for latent variable analyses explicitly encoding batch, sample, and cell events as separate dimensions, but this has not yet been implemented for mass cytometry data.

Currently, no readily available method exists specifically for batch adjustment of mass cytometry data. As the number of cell events measured for each sample may be in the hundreds of thousands to over a million, with 20 samples per batch, and likely many batches per study, a computationally efficient method that will execute in a reasonable amount of time without specialized computing hardware is needed. Another feature incorporated into the design of this batch normalization method is a simple conceptual interpretability, in which, similarly to bead normalization, the resulting files are adjusted to address variability and the output is a ready to analyze Flow Cytometry Standard (FCS) file (21). Additionally, an optional diagnostic output allows for user-driven evaluation of the batch normalization of their dataset. Finally, a “rolling basis” capability to adjust individual batches as they become available is desirable, as it allows for inclusion of additional batches without reprocessing or changing the results for batches already completed.

To facilitate comparison across batches, it is useful to include a technical replicate in each barcode set as a biologically constant reference, or anchor sample. This anchor sample represents a single donor/cell line/tissue sample (depending on the goal of the study) that is processed in the same way as the other study samples, and distributed in aliquots to be incorporated into each barcode set. Additionally, if the study design involves *in vitro* stimulations to induce specific functional read outs, anchor samples may need to be prepared as an unstimulated and stimulated pair (or more than a pair if the study design calls for it). A “master set” of unstimulated and stimulated anchor samples can be prepared prior to the start of the project, and distributed in single aliquots to later be included in each barcode set. While this approach reduces the number of study samples per barcode set, using the same anchor sample across all batches minimizes biological differences, isolating batch effects. Rather than estimating the effects per batch based on modeling all samples, which requires assumptions such as homogeneous sample composition across batches (18, 22), batch effects for each channel (measured variable) can be directly estimated from the anchor samples, where the expectation of homogeneity in cell composition and marker expression levels is much more reasonable. Scaling or mapping parameters per channel for an anchor sample may then be applied to other samples from the same batch.

Data standardization based on location and scale adjustment are well-characterized and widely used (23). These include combinations of spread-based methods for adjusting variance (autoscaling, Pareto scaling) and magnitude-based methods such range scaling, level scaling (mean), and median or percentile matching. Here we describe a flexible method to adjust mass cytometry batches relative to a reference batch based on technical replicates, implemented in R, and available at https://github.com/CUHIMSR/CytofBatchAdjust. We implement several standard normalization options, and provide recommendations for their use. Available methods include per channel quantile normalization (QN), and location and scale methods that harmonize batches based on signal variance, mean, median, or a user-defined percentile. We note that in channels with high variability among technical replicates, particularly those with substantial variability in the fraction of zero-valued events, there may be no satisfactory adjustment to harmonize data across batches. In these cases, it may be advisable to conduct statistical testing for conditions of interest within batches, then combine results across batches using Fisher's method as recommended by others (24).

To evaluate methods and parameter settings for batch adjustment, we quantify cell subpopulations using both manual gating and unsupervised clustering, applied to multiple technical replicates run in different batches, before and after adjustment. We also quantify variability in signal intensity of cytokines measured after stimulation. As each anchor is a large random sample from the same initial population of cells, we expect the same proportions of cell types in each batch anchor. Therefore, lower variability in subpopulation frequency (percentages) and cytokine levels (signal intensity) among technical replicates after adjustment indicates successful reduction of batch effects. Decreased variability in a second set of replicate samples (i.e., stimulated samples from an anchor set) demonstrates that batch adjustment parameters based on an anchor sample are applicable to the batch that it represents.

## Batch Normalization Method

An example dataset of 12 barcode sets is be used to illustrate the development of this batch adjustment methodology, its validation and application. Data was acquired on a Helios CyTOF instrument (Fluidigm, San Francisco, CA) over the course of 6 months. In this data set, 38 markers (22 cell surface markers and 16 intracellular cytokines) were used to study immune dysregulation in pediatric systemic lupus erythematosus (SLE). In addition to the study samples, a pair of anchor samples was included in each barcode set (Cell-ID^TM^ 20-plex barcoding kit, Fluidigm, San Francisco, CA). The anchor samples were generated from one single healthy donor peripheral whole blood sample, processed to include an unstimulated and a stimulated conditions (LPS + R848) to induce the 16 cytokines measured, as previously described in O'Gorman et al. (3). These samples were used to demonstrate the applicability of this adjustment methodology and validate the decrease in total variance across anchor samples post-adjustment (see Supplementary Material for the specific FCS files that were used, http://flowrepository.org/id/FR-FCM-Z2YR). This dataset was acquired on a Helios CyTOF instrument at Stanford University over the course of 4 months. Peripheral blood mononuclear cells (PBMCs) from a healthy control were spiked into separate barcoded samples and stained with a panel of 38 surface markers, as part of a longitudinal vaccination study. There were no stimulation conditions used in this dataset. The healthy control files (anchors/references) were not used further in the analysis of the study samples.

In Kleinsteuber et al. (25) the use of reference samples is also applied, albeit taking a different approach—CD45-barcoded anchor sample aliquots were spiked into each individual study sample, which were run sequentially (separately) across the study. This reference sample was then used to guide manual adjustment of gates. In contrast, in the approach described here, the reference sample (stimulated anchor) from each batch was then used as a representative of that batch to determine adjustment factors or mapping functions (per channel and per barcode) that bring the anchor samples into alignment. This adjustment was automated and computed for each barcode set and each channel, and independently applied to all samples from the same barcode set on a per channel basis, including the unstimulated anchor samples which served as a validation set.

**Figure 1:**
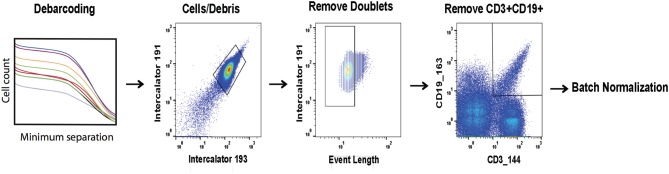
Pre-processing of files prior to batch normalization. FCS files are bead normalized and debarcoded. A set of three consecutive gates is applied as shown prior to batch normalization.

Prior to batch adjustment, data was bead-normalized (15), debarcoded (11), and manually gated to remove debris and non-biological events (Figure 1). Alternatively, data pre-processing could be automated using FlowClean or other methods (26). For batch adjustment, a single stimulated anchor sample from a specific barcode set was designated as reference. The reference anchor may be chosen by any criterion. We recommend visual inspection of data distributions to avoid choosing an outlier batch as reference. All batches were adjusted relative to this reference, and samples from the reference batch were not modified. Importantly, signal intensity range in anchor samples used for adjustment should approximately cover the full signal range for all samples, and therefore should include experimental manipulations. We also evaluated an unstimulated anchor replicate set for validation to demonstrate that adjustments computed using the stimulated anchors are also applicable to the unstimulated samples.

Using the stimulated anchor samples from each of the 12 barcode sets, for each batch and each channel independently, an adjustment factor was computed as a function of the ratio of a descriptive summary statistic for that channel and anchor to the same value for the reference anchor sample. We implemented and tested several batch adjustment options, including scaling by mean and median [similar to bead normalization (15)], standard deviation of per event ion counts, and quantile normalization (QN), which was developed for oligonucleotide arrays and is used to normalize RNA-seq data (27, 28). Additionally, a user-defined percentile may be specified rather than median. This approach is useful in the case that any channel has more than 50% zero-valued events (from total events per file after data pre-processing steps as described in Figure 1), giving a median of zero and an undefined or zero-valued scaling factor. The same adjustment approach (i.e., mean, median, standard deviation, QN, percentile scaling) is applied across the entire dataset, and the same approach is applied to all channels, though each adjustment factor is tailored to each barcode set and channel. Adjustment factors may be computed and applied in an arcsinh-transformed space commonly used for data visualization, or in the untransformed ion count data space. Scaling in the transformed data space effectively results in a non-linear adjustment once the inverse transformation is applied before results are written to the resulting FCS file, whereas adjustments computed in the original data space are linear.

We began by evaluating distances between distributions for all individual data channels among replicates for a set of candidate parameter combinations using the Kolmogorov–Smirnov distance metric. Using parameters that performed well in single channel consistency measurements, we then moved to evaluations considering all channels together, and finally to consistency of subpopulation frequencies simultaneously for 12 cell types in all replicates. Levine et al. scaled individual data channels to the 99.5th percentile before defining subpopulations, and Lun et al. suggested a range-based batch adjustment to linearly scale intensities between the 1st and 99th percentiles (29, 30). We used the 95th percentile as the high end for our normalization target point to avoid outliers, and 80th percentile as the low end, as up to 79% of cell events were zero-valued for some channels in this data set. Using default parameters, the adjustment factor for each channel was computed as the ratio of the 95th percentile event ion counts for each batch anchor compared to the designated reference anchor. Adjustments were made in the untransformed raw ion count data space. We found that these parameters resulted in the most stable post-adjustment population counts using the validation method described below.

Execution time scaled linearly with the number of samples. Using a Mac Mini (Apple, Cupertino, CA) with a 2.8 GHz processor requires ~8 s per sample, including time to read the input file, apply the adjustment, and write the result to disk. A log file was generated listing each batch found, each sample processed, and additional diagnostic information, including processing time. Adjustment factors for each channel in every batch (or mapping functions in the case of QN) were saved in a .Rdata file (31). An optional diagnostic output can be generated, which includes (1) the scaling factors for every channel for each barcoded anchor that is adjusted, and (2) the signal intensity distribution for each adjusted channel for each barcoded anchor pre- and post-adjustment (from total events per file). We recommend inspecting adjustment factors, as very large values may be undesirable, and could result from outlier batches, channel labeling errors, or other issues with input data.

## Batch Normalization Validation

All anchor samples, including the stimulated and unstimulated sets, were derived from aliquots of a single large sample and processed together to minimize variability. Whereas the true numbers of cells of any phenotype in the anchor samples is unknown, given the large number of cell events measured for each anchor aliquot (300–500 k events per anchor per batch), subpopulation frequencies are expected to be consistent among anchors. Therefore, variation in population frequencies among anchor samples is expected to be attributable to technical effects, and should decrease after batch adjustment. With similar reasoning, we expect the functional responses in the stimulated anchor relative the unstimulated condition to be consistent across anchor replicates.

### Single Channel Consistency Measures

As an initial evaluation comparing methods and parameters, we used the Kolmogorov–Smirnov statistic to measure the distance between distributions among replicates within single channels. Within each channel, pairwise empirical distribution distances were computed between each pair of replicates and averaged, giving a single measure of consistency for each data channel across all batches of the experiment. This test was automated and less labor intensive than the manual gating evaluation described below, allowing an expedient exploration of a range of approaches and parameter settings.

For a comprehensive measure of signal variability, total variance was computed as the sum of the eigenvalues of the covariance matrix (32) of the mean signal intensity for all cell events in each channel and each replicate. The value of the total variance is equal to the sum of the diagonals of the covariance matrix, i.e., the sum of the individual variance components. The significance of the decrease in total variance due to batch adjustment was evaluated using a permutation test (33), where the labels pre- and post-adjustment were swapped for each replicate. Change in total variance was computed for all possible permutations to derive a null distribution for comparison with the observed reduction in total variance in signal intensity (Figure S1).

As the goal of a batch adjustment procedure is to prepare the data for further downstream analysis, a more important measure than single-channel distribution distances and mean signal intensity variability is consistency in cell subpopulation frequencies among replicates. These two evaluation approaches will not always agree because gating is hierarchical and considers multiple channels simultaneously. We show that this is the case for some parameter settings, which performed well within channels, but did not translate to better performance in cell-type classification consistency.

### Cell Subpopulation Frequency Quantification Consistency Measures

Two methods to assess the consistency of cell subpopulation frequencies across anchor samples were used: a consistent manual gating strategy applied to all anchors (no tailored gates), and unsupervised population quantification via event clustering considering all anchors simultaneously. As functional read outs, cytokine production was assessed, either as percent positivity or mean signal intensity, comparing unstimulated and stimulated anchors within the relevant gated parent populations. Although the normalization procedure applied to the data did not specifically adjust pre-defined cell populations, the adjustment and subsequent reduction of variability in signal intensity improved downstream manual gating procedures by reducing the need to manually tailor gates.

### Manual Gating

The stimulated anchor sample designated as reference was manually gated using FlowJo (version 10) for the cell populations defined by the markers shown in Table S1. This set of gates was applied without manual adjustment to all pre- and post-normalized anchor samples separately, and each cell population frequency was quantified as the fraction of all cell events within a sample. As a further validation, unstimulated anchors were also examined. By quantifying populations in unstimulated replicates that were adjusted using the stimulated anchor samples we showed that this method is applicable to a range of samples and experimental conditions. Additionally, signal intensity for functional markers within specific manually gated populations was also compared pre- and post-batch normalization.

To quantify the magnitude of cell population frequency variability for an entire experiment we used the total variance defined as the sum of the eigenvalues of the covariance matrix of all gated subpopulation fractions in all replicates. Similar to the test of signal intensity variability described above, the difference in total subpopulation variance pre- and post-adjustment provides a measure of the effectiveness of the adjustment, and a means to compare methods and parameter settings. To assign a significance level to the change in total population fraction variance, we again used a permutation test, where labels of replicate samples were swapped between pre- and post-adjustment conditions for each replicate. The change in total variance was then recomputed for all possible permutations, giving an exact test of the probability of seeing a reduction in variance of population frequencies as or more extreme than that observed for the unpermuted data (Figure S1).

### Unsupervised Population Quantification

**Figure 2:**
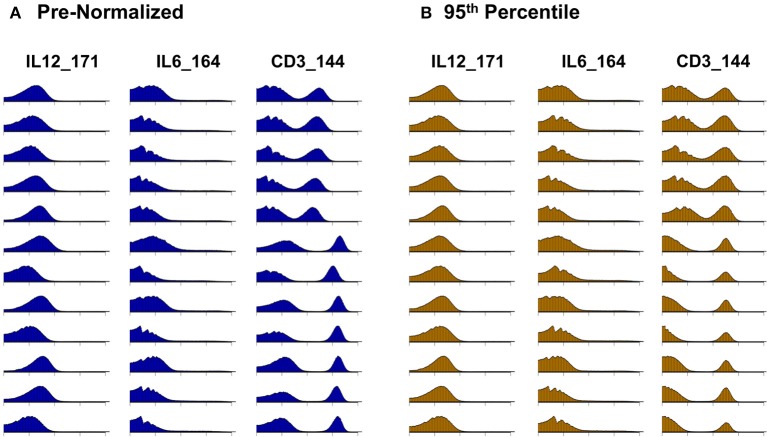
Signal intensity distribution pre- and post-batch normalization. Density plots for all barcode anchor replicates for three representative data channels (IL12, IL6, CD3). **(A)** Before batch normalization. **(B)** After batch normalization to 95th percentile, relative to anchor 1.

Citrus version 0.8 (34) was chosen as the unsupervised clustering approach to explore differences between pre- and post-normalized files. Samples were grouped as pre- or post- normalized, with 50,000 events sampled from each file and a minimum cluster size of 2.5%. The significance analysis of microarrays (SAM) statistical model was used to examine statistical differences with a false discovery rate (FDR) below 1%. In addition to quantitative evaluation, visual inspection of data distributions per channel across anchor samples provides a qualitative assessment of pre- and post-adjustment consistency and the effectiveness of batch adjustment (Figure 2).

## Batch Normalization Results

### Signal Intensity Variability and Zero-Valued Events

**Figure 3:**
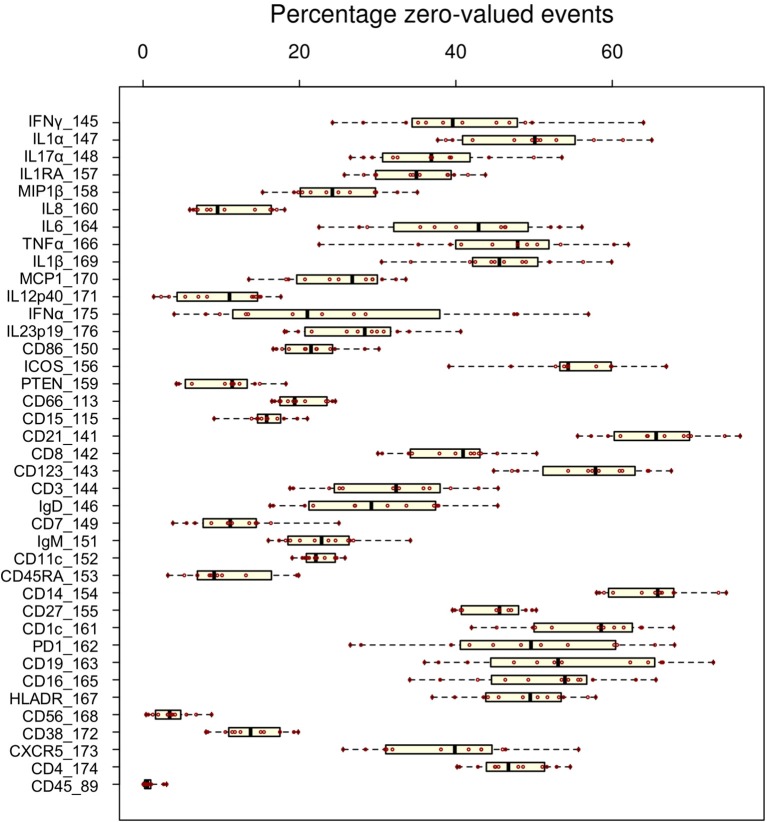
Variability of zero-valued events among replicate anchor samples pre-normalization. The fraction of zero-valued events (X-axis) for each anchor (red dot) is shown for each channel (Y-axis).

**Figure 4:**
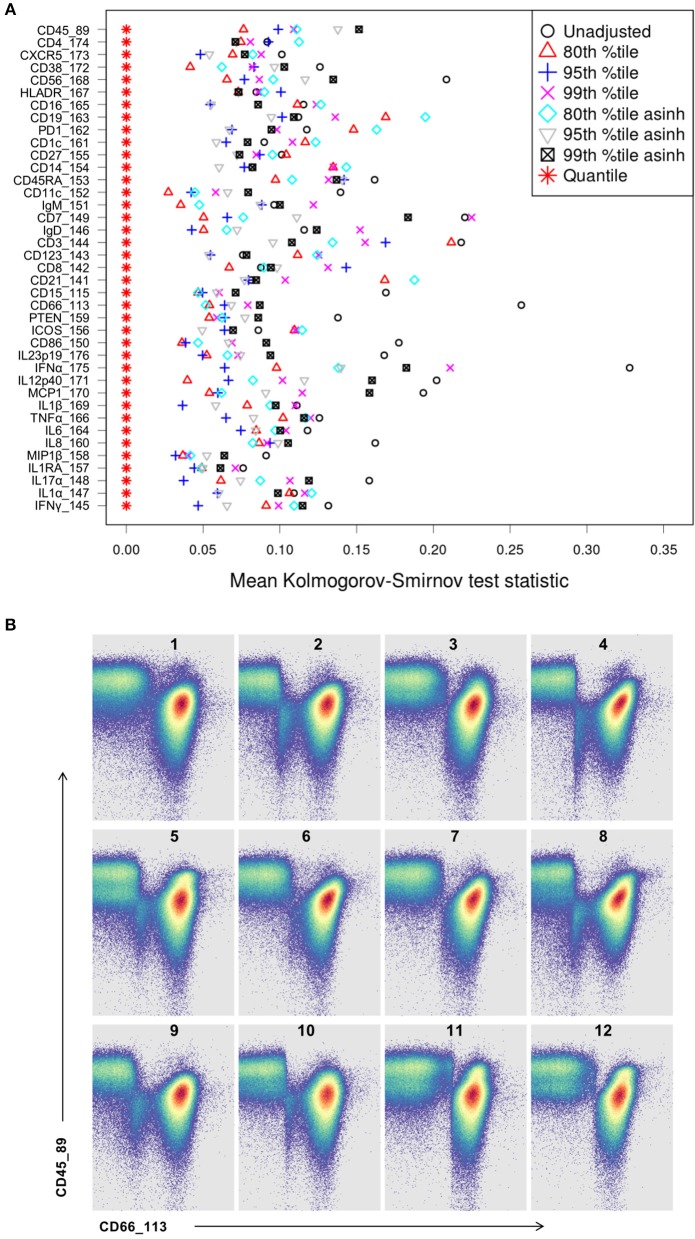
Kolmogorov-Smirnov (KS) distance for each single channel, for each batch normalization approach examined. **(A)** Distance between distributions among replicates is computed as the average of the KS test statistic for all pairwise combinations of anchor samples, computed independently (X-axis) for each data channel (Y-axis). Smaller values indicate better consistency among replicates. **(B)** Quantile normalization artifacts in biaxial plots for each anchor replicate (numbered). Top left is the unmodified reference anchor.

Whereas some channels are quite consistent across batches, others show substantial variability (Figure 2A). In addition to shifts in signal density and changes in distribution shape (number of visible peaks), we observed large differences between replicates in the number of events within a mass channel with ion counts of zero (Figure 3, Figure S2). The differences in the numbers of zero-valued events are particularly troublesome for quantile normalization, which either inflates the zeros to non-zero values introducing noise, or squashes the lowest non-zero events to zero, resulting in loss of information. Further, given large initial differences among distributions, we found that adjusting each channel independently using quantile normalization introduced visual artifacts when viewed as biaxial plots in the process of manual gating. Although quantile normalization can enforce identical distributions among replicates and therefore produces the most consistent results on the single-channel Kolmogorov–Smirnov test (Figure 4A), we excluded this option from further analysis of cell population fraction variability due to the artifacts it introduces (Figure 4B).

**Figure 5:**
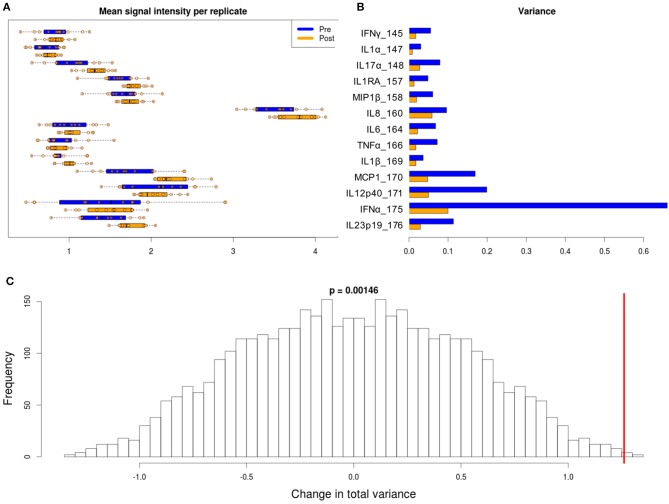
Variability in signal intensity per channel for the 95th percentile batch normalization approach. **(A)** Mean signal intensity of each cytokine for all cell events for each anchor replicate (circles). **(B)** Variance of these mean signal intensities across replicates. **(C)** Permutation null distribution and significance of observed change in total variance (red line). *p*-value is the fraction of permutations with a change in total variance as or more extreme than that observed for unpermuted data.

**Figure 6:**
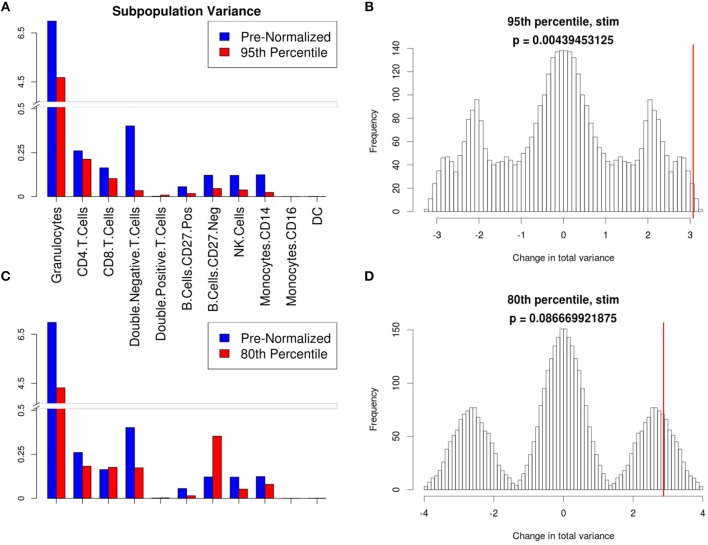
Manual gating cell subpopulation variance post-normalization. Variance across all stimulated anchor replicates in the fraction of events assigned to each population pre- and post-normalization. Manual gates were drawn based on the reference anchor (barcode 1) and applied to all other anchor replicates. **(A,C)** Variance per subpopulation pre- (blue) and post-normalization (red) for 95th **(A)** and 80th **(C)** percentile normalization. **(B,D)** Null distribution for change in total variance for the 95th **(B)** and 80th **(D)** percentile normalization. Red bar indicates observed change in total variance. *p*-value is the fraction of permutations with a change in total variance as or more extreme than that observed for unpermuted data.

Several combinations of parameters resulted in decreased distances among empirical distributions within single channels (Figure 4A). Based on these results and visual inspection of distributions and gated populations, percentile-based adjustments made in the raw (untransformed) ion count data space appeared most effective, and 80th and 95th percentile scaling were chosen for further evaluation. Both options substantially reduced per-channel signal variability (Figure 5, Figure S3) and manually gated subpopulation variance (Figure 6, Figure S4), as described below.

### Manual Gating Results

**Figure 7:**
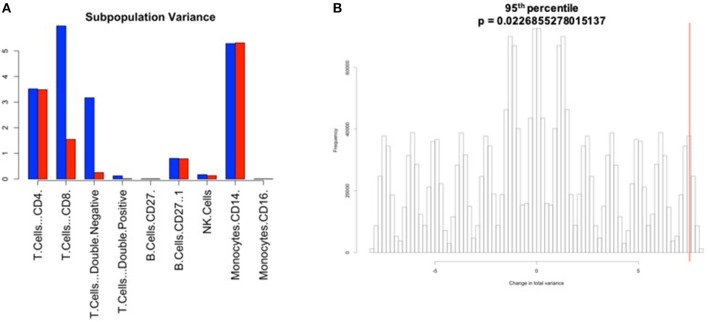
Manual gating cell subpopulation variance post-normalization. Variance across all stimulated anchor replicates in the fraction of events assigned to each population pre- and post-normalization. Manual gates were drawn based on the reference anchor (barcode 1) and applied to all other anchor replicates. **(A)** Variance per subpopulation pre- (blue) and post-normalization (red) for 95th percentile normalization. **(B)** Null distribution for change in total variance for the 95th percentile normalization. Red bar indicates observed change in total variance. *p*-value is the fraction of permutations with a change in total variance as or more extreme than that observed for unpermuted data.

**Figure 8:**
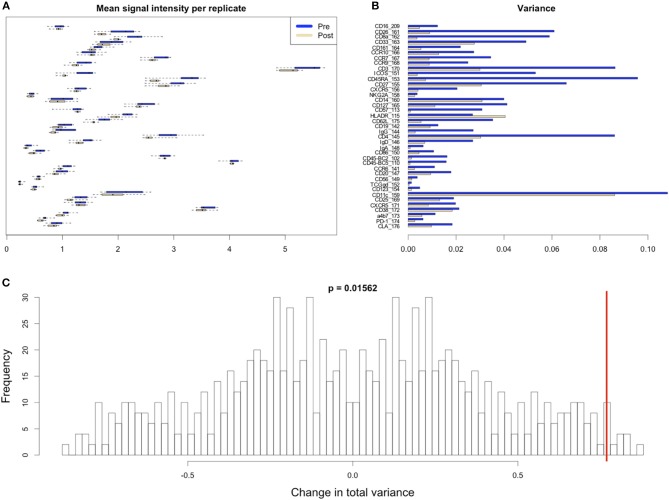
Variability in signal intensity per channel for the 95th percentile batch normalization approach. **(A)** Mean signal intensity of each cytokine for all cell events for each anchor replicate (circles). **(B)** Variance of these mean signal intensities across replicates. **(C)** Permutation null distribution and significance of observed change in total variance (red line). *p*-value is the fraction of permutations with a change in total variance as or more extreme than that observed for unpermuted data.

With a set of gates manually defined using the stimulated reference anchor and applied to all anchor samples, variance in subpopulation proportions among replicates was decreased after batch adjustment. Decrease in total variance of cell subpopulation frequencies for each of the parameter combinations above is shown in Figure 6. These results indicate that scaling based on the 95th percentile in the original ion count (untransformed) data space was the most effective parameter combination for batch adjustment for this dataset. This same 95th percentile scaling batch adjustment method was applied to the additional dataset from the publicly available flow repository, also demonstrating a decrease in total variance for both frequency of manually gated populations (Figure 7) and per-channel signal intensity variability (Figure 8).

**Figure 9:**
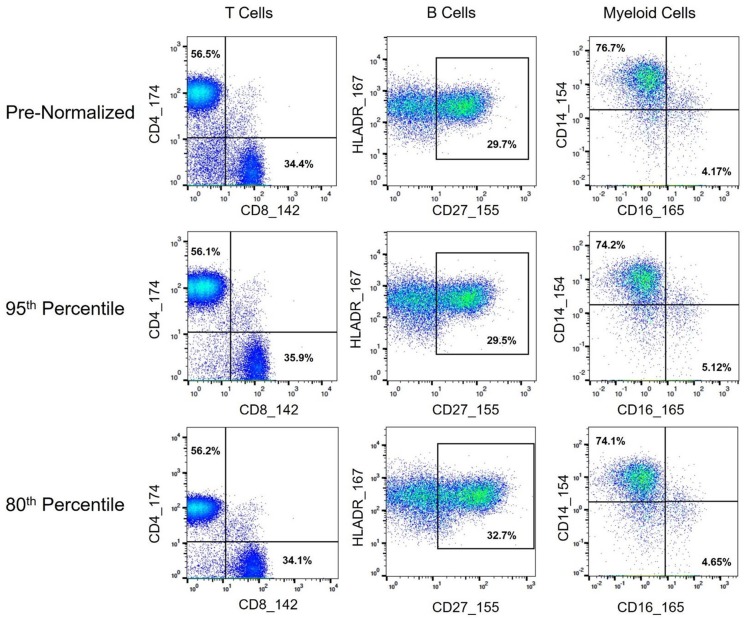
Immune cell subset frequencies pre- and post-normalization, assessed via manual gating. Biaxial plots and population percentages (percent of parent gate) for three immune cell subsets across representative pre- and post-normalized anchor sample (80th and 95th percentile shown). Population percentages correspond to CD4 vs. CD8 T Cells (CD45+, CD3+, CD19–, and CD4+ or CD8+, respectively), CD27hi B Cells (CD45+, CD3–, CD19+, HLADR+, CD27hi), and CD14 vs. CD16 Monocytes (CD45+, CD3–, CD19–, HLADR+, CD11c+, and CD14+ or CD16+, respectively).

**Figure 10:**
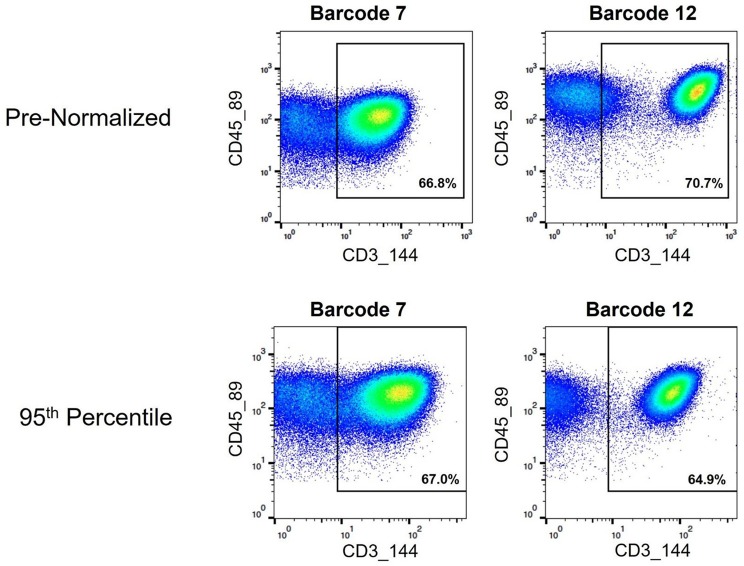
Assignment of cell events to CD3+ T cell gate pre- and post-normalization. Biaxial plots showing cell events that fall within a CD3+ gate used for examining T cell subsets. Two pre- and post-normalized anchor samples are shown.

Granulocytes and other lymphoid and myeloid populations of interest (T and B cell subsets, NK cells, monocytes, and dendritic cells) were examined to confirm that the batch normalization procedure did not alter standard manual gating and downstream analyses, and also to confirm that the procedure did not disproportionately affect certain populations relative to others. In addition to the populations examined above, gates to examine double negative (CD4–/CD8–) and double positive (CD4+/CD8+) T cells were drawn to help elucidate potential batch effects. The frequency of double negative T cells was greater in the pre-normalized group relative to the batch normalized group (*p*-value: 0.0005 *t*-test), indicating the presence of batch effects since the cells that should have been categorized as non-T cells (CD3–) were incorrectly included within the T cell gate (Figure 9). In addition to the decrease in variance for the batch-normalized populations, the reduced misclassification of CD3– cells further supported the conclusion that the normalization procedure reduced batch effects (Figure 10). Gating of the unstimulated anchors served as a secondary means of validation with similar results (Figure S4).

**Figure 11:**
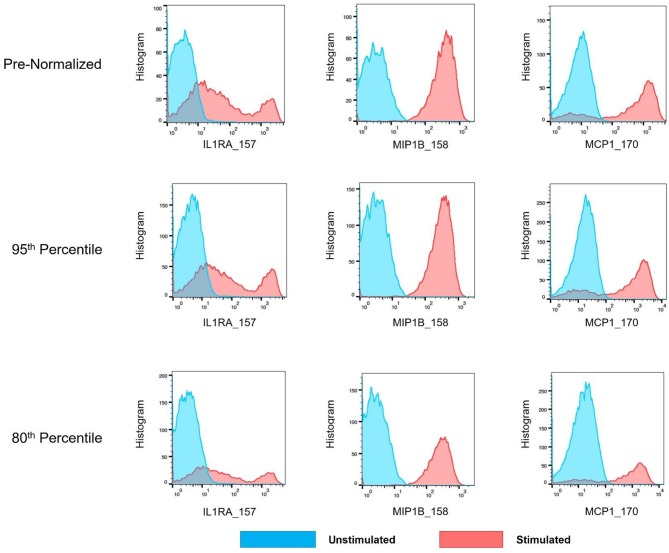
Cytokine signal intensity distribution in unstimulated and stimulated anchor samples pre- and post-batch normalization. Histograms showing signal intensity for three cytokines for the CD14^hi^ monocytes, in paired unstimulated and stimulated anchor samples, pre- and post-normalization (95th and 80th percentile).

As mass cytometry is often used to address questions related to cellular functionality under stimulated conditions, intracellular cytokine markers were examined to assure the batch normalization process does not add unnecessary noise. Three functional markers (IL1RA, MIP1B, and MCP1) were examined within CD14^hi^ monocyte populations (CD45+, CD3–, CD19–, CD7–, HLADR+, CD11c+, CD14+, and CD16–) across unstimulated and stimulated anchor samples. Histograms and exported means from FlowJo indicated that the application of the batch normalization method did not eliminate the expected functional responses (i.e., cytokine production) from populations of interest, but yet adjusted the signal intensity for the mean expression values according to the adjustment factor calculated from the normalization process (Figure 11, Table S2).

### Clustering Results

**Figure 12:**
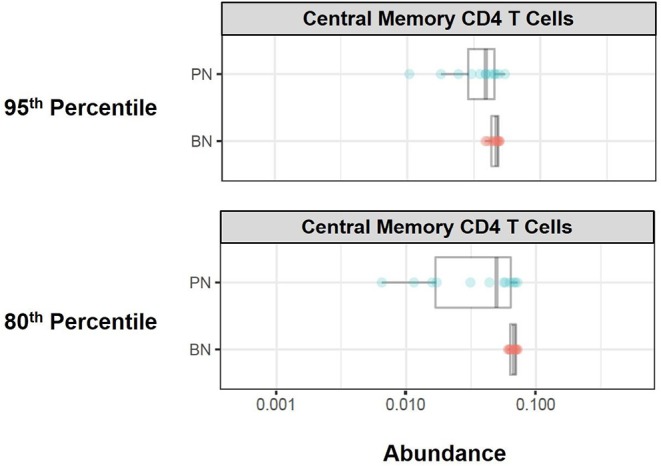
Immune cell subset frequencies pre- and post-normalization, assessed via unsupervised citrus analysis. Citrus was applied using PAMR analysis with false discovery ratio of 0.05. Boxplots of relative abundance for central memory CD4 T cells generated from Citrus pre- (PN) and post-normalization (BN, 95th and 80th percentile).

Citrus Significance Analysis of Microarrays (SAM) analysis revealed one particular T cell subpopulation from the batch-normalized group that had significantly greater abundances relative to the pre-normalized samples (Figure 12). These clusters were determined to be CD4 Central Memory T Cells (CD3+, CD4+, CD27+, and CD45RA–). Of note, the variance in these clusters for the batch-normalized files was remarkably reduced relative to the pre-normalized files.

### Batch Adjustment Diagnostic Output

Batch adjustment approaches and best strategies are likely to differ based on the nature/qualities of the dataset. For this reason, investigators are encouraged to explore different batch adjustment approaches available through the software package described. To facilitate this evaluation process, we have added a graphical diagnostic output for percentile-based batch adjustments depicting the relative sizes of the scaling factors used for each batch within each data channel. Investigators may quickly identify outliers from this figure and decide whether or not they wish to eliminate certain batches that are outliers (Figure S5). For further inspection, we have also added signal intensity distribution plots for every channel, for every anchor sample from every batch pre- and post-adjustment, with one figure generated each data channel (Figure S6). Using the scaling factors figures and the signal distribution figures, users may determine whether to exclude any batches from further analysis. An R data file is also saved containing scaling factors for each batch for more detailed examination.

## Discussion

Mass cytometry has the potential to reveal relevant differences in cell type composition and function across multiple immunological pathways, aiding in biomarker discovery and mechanistic hypothesis generation. Methods testing for differential cell subpopulation abundance or marker intensity assume that intensities are comparable across samples (30). Sample barcoding and pooling largely addresses this issue among samples within a batch, but make no adjustment for systematic differences between batches (11). Bead normalization (15) corrects for machine drift occurring over the course of a single experiment or across multiple experiments, but does not address other aforementioned technical issues contributing to batch effects—variability in reagent lots, instrument maintenance and calibration, and antibody concentration to cell number ratio per barcode amongst others.

Quantile normalization (QN) is an attractive approach to batch adjustment, as it forces all samples to fit the same target distribution regardless of the shapes (properties) of their starting distributions, and several variations have been developed for the analysis of RNA-seq data (35). Indeed, by both visual inspection and the Kolmogorov-Smirnov metric of inter-distribution distances, QN appears quite effective for single channels considered in isolation. However, here we have shown that the potentially large changes introduced by single-channel QN can introduce artifacts when viewed in two-dimensional plots. Other methods for handling batch effects rely on the assumption of sample homogeneity among batches (16). Careful experimental design requires allocating an equal number of a given condition to each batch to avoid confounding. With large enough batches (hundreds to thousands of samples, depending on the data characteristics) this assumption may be approximately valid, as outliers or variability within conditions being studied may average out with large numbers of samples. Current mass cytometry barcode sets typically consist of 20 samples. With three experimental conditions (e.g., drug, stimulation, untreated), the effective number of individuals composing a batch may drop to six, increasing the likelihood that a single outlier individual could skew a batch, violating the assumption of homogeneous composition between batches. There is substantial variability of immune system composition and function even within the healthy population, and outliers are expected in potentially heterogeneous disease conditions. This inherent variability and the small size of mass cytometry batches make assumptions of homogeneous batch composition questionable.

To address these issues, we describe the use of technical replicates included in each barcode set as reference points to anchor each batch. Adjustments are calibrated using anchor samples representing each barcode set, then applied to all samples composing a batch. This strategy avoids any batch composition assumptions, while comparing identical anchor samples eliminates biological variability and isolates the technical effects particular to each batch. For validation, we use a second set of (unstimulated) replicate samples in each batch, that are adjusted along with the other samples using parameters determined with the representative anchors. Significant reductions in variability across batches in the stimulated anchor replicates, validation of this variability decrease in the unstimulated anchor samples, and the use of statistical measures to evaluate the reduction of this variability, demonstrate the feasibility and effectiveness of a normalization strategy via which an entire batch is adjusted using parameters derived from representative anchors in each barcode set.

Despite the multitude of existing mass cytometry data analysis tools, challenges in choosing the appropriate analytical strategy to answer the relevant biological question still remain—What is the appropriate algorithm to use for the study? Should one or multiple algorithms be applied to analyze the data? If more than one analytical method is used, how will results be integrated? What is the best way to go back to the raw data from the unsupervised analysis result? These questions remain a significant challenge that often discourages the application of mass cytometry analysis to human immunological studies, where single statistically meaningful outcomes are expected for clinical application. These analytical barriers are further compounded by the fact that human clinical studies often require prospective/longitudinal timeframes and multiple batches; and the lack of a batch adjustment/normalization method for mass cytometry data poses an added (if not crucial) hurdle to downstream analysis of human clinical studies. Here we describe the development, statistical validation, and application of an automated batch adjustment tool designed to minimize batch effects and allow for relevant downstream analysis. This novel tool is time efficient, incorporates flexibility for data types, investigator-driven batch adjustment approach choices, and the ability to evaluate such adjustment approaches (optional diagnostic output)—with great application potential for the analysis of large samples in human clinical studies.

## Materials and Methods

### Study Approval

Human samples were obtained from the Allergy and Immunology Clinic at Children's Hospital Colorado. Age appropriate consent and assent was obtained. All human donors were enrolled under study protocol 16-0918, approved by the Institutional Review Board of the Research Compliance Office at University of Colorado. Stage III melanoma patients were recruited at the University of Colorado Cancer Center Cutaneous Oncology Clinic as part of the clinicaltrials.gov registered clinical trial NCT02403778. All patients provided a written informed consent, and the treatment protocol was approved by the Colorado Multiple Institutional Review Board (#14-0948).

### Mass Cytometry Analysis

SLE patient, gender-matched control, or healthy donor (anchor) peripheral whole blood was collected into heparinized vacutainers (BD). For CyTOF analysis, blood samples were fixed with Phosflow lyse/fix buffer (BD 558049) either immediately after collection (T0); or after incubation at 37C, mixed 1:1 with RPMI 1640 (Gibco 21870076) plus protein transport inhibitor cocktail (eBioscience 00-4980-93), and with R848 (1 μg/ml; Invivogen tlrl-r848) for 6 h (T6). Lysed/fixed cells were stored at −80°C, and were thawed on the day of barcoding and staining. To decrease technical variability, palladium isotopes were used in different combinations for mass tag barcoding of separate samples, pooled in sets of 20, surface stained in a single tube with a metal-labeled antibody panel, then permeabilized with Perm/Wash buffer I (BD 558050) to facilitate intracellular staining. Barcoding methodology was adapted from Zunder et al. (11). Protocols for intracellular cytokine staining (ICS) assays were adapted from previous studies in O'Gorman et al. (3).

## Data Availability Statement

R code and usage instructions are available at: https://github.com/CUHIMSR/CytofBatchAdjust. The current implementation runs on linux or mac platforms.

## Ethics Statement

The studies involving human participants were reviewed and approved by Institutional Review Board of the Research Compliance Office at the University of Colorado study protocol 15-2211. The patients/participants provided their written informed consent to participate in this study.

## Author Contributions

RS and CJ: methodology and resources, validation, formal analysis, investigation, and writing original draft. RS, CJ, JG-P, RB, and SO: writing-review and editing. RR, DG, PR, and EH: supervision, project administration, and funding acquisition.

### Conflict of Interest

The authors declare that the research was conducted in the absence of any commercial or financial relationships that could be construed as a potential conflict of interest.
