# Supplementary material for: Minimizing Batch Effects in Mass Cytometry Data
Source: Front Immunol. 2019 Oct 15;10:2367. doi: 10.3389/fimmu.2019.02367 (PMC6803429; doi:10.3389/fimmu.2019.02367)
Supplement: Supplementary file 1 [file Data_Sheet_1.PDF]

frontiers

Supplementary Material

Supplementary Figures

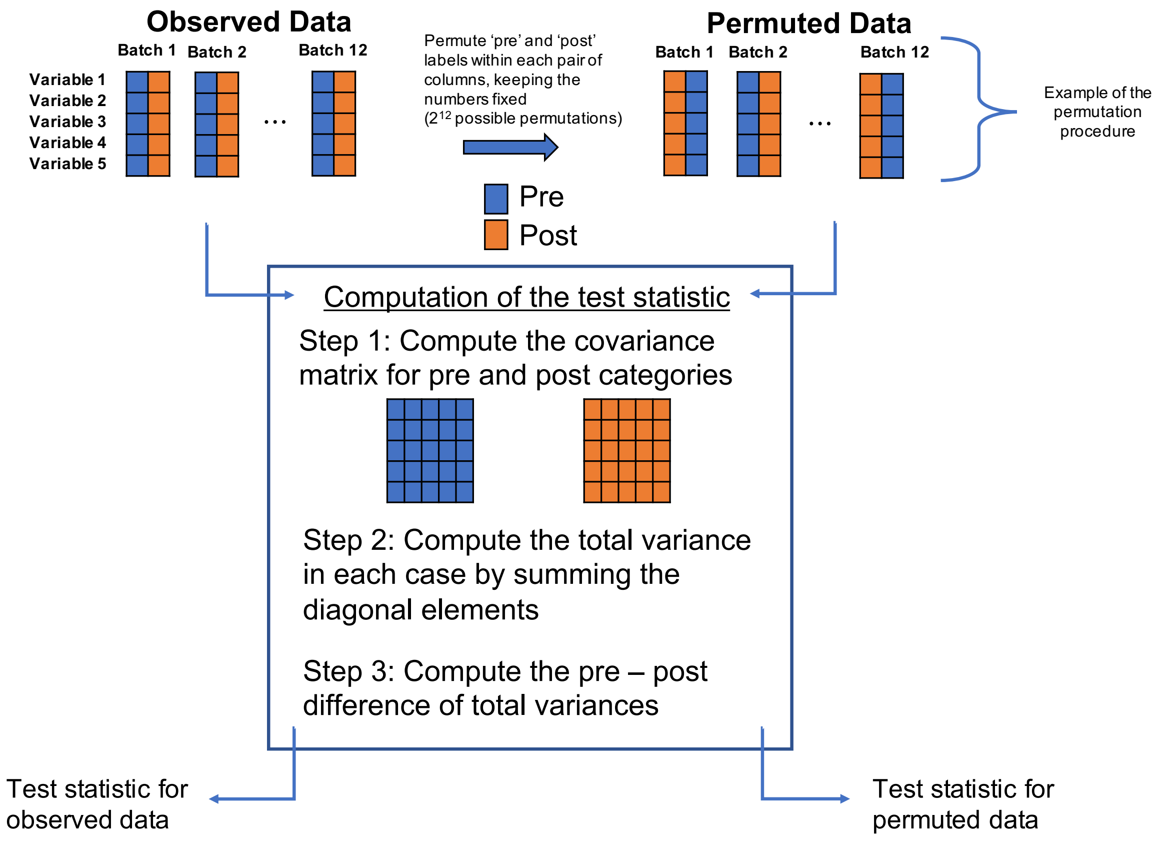

Supplementary Figure 1. Permutation test illustration. Illustration of the permutation procedure and calculation of the total variance difference for data with 5 variables and 12 batches. The variables can be either proportions of different cell types or mean signal intensity of different cytokines.

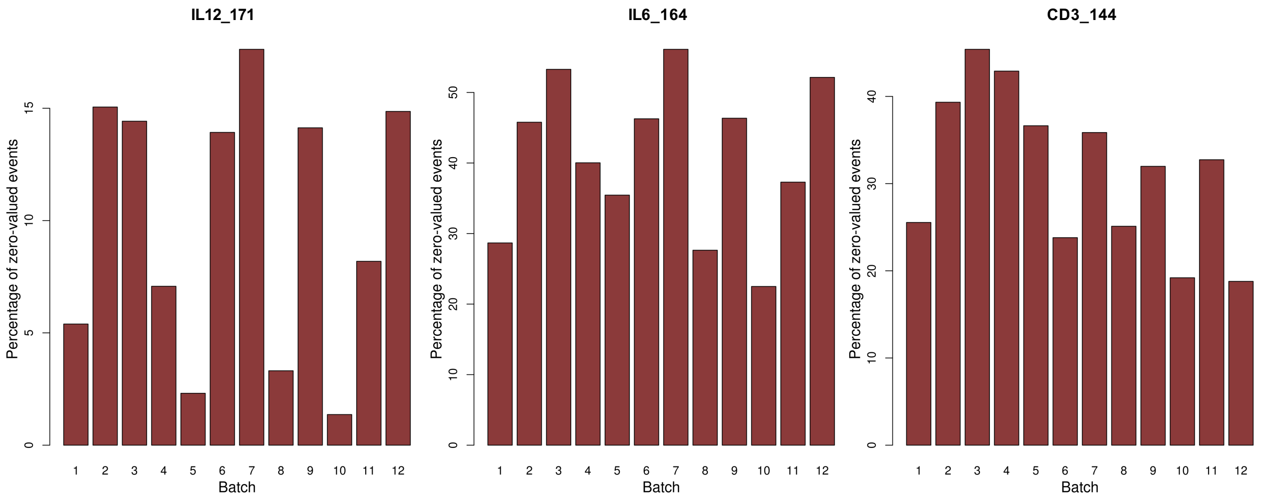

Supplementary Figure 2. Percentage of zero-valued events for specific channels. Bars show the percentage of zero-valued events in each barcode anchor for three parameter_channels.

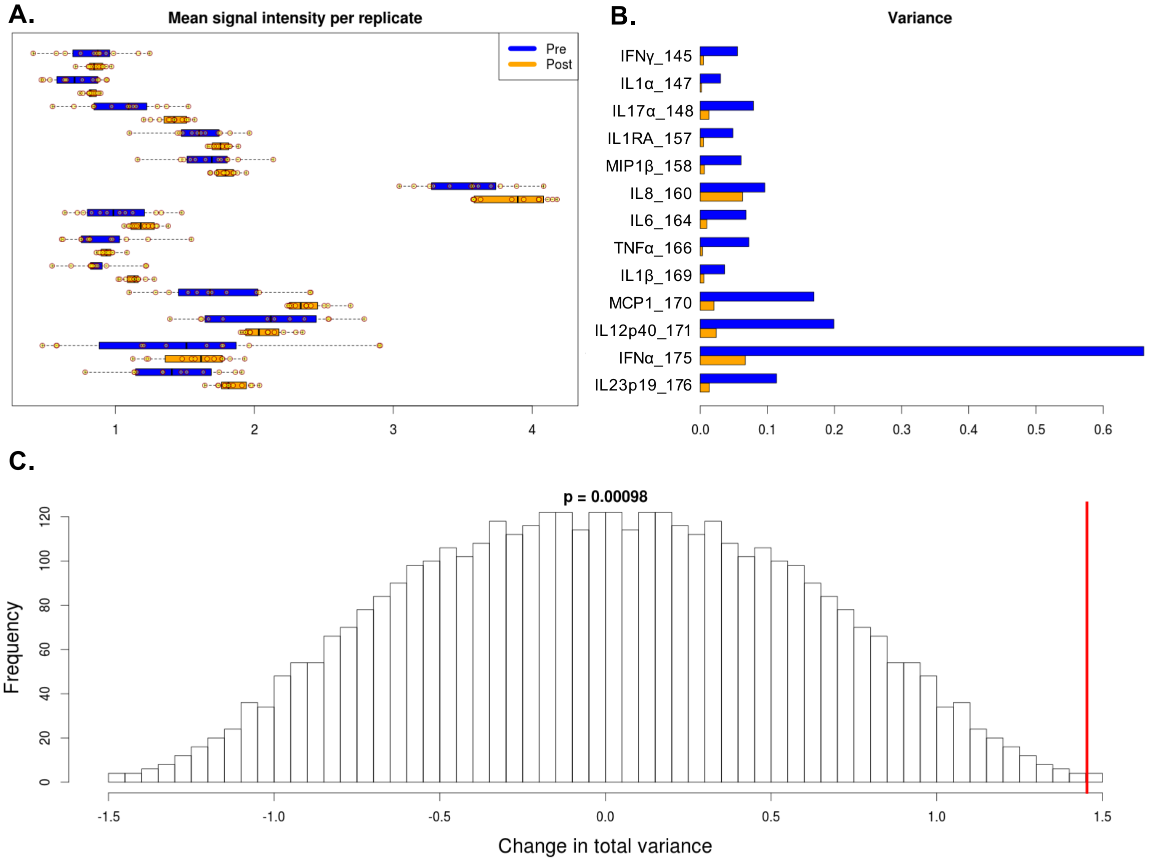

Figure S3. Variability in signal intensity per channel for the 80th percentile normalization. A. Mean signal intensity of each cytokine for all cell events for each anchor replicate (circles). **B.** Variance of these mean signal intensities across replicates. **C.** Permutation null distribution and significance of observed change in total variance (red line). p-value is the fraction of permutations with a change in total variance as or more extreme than that observed for unpermuted data.

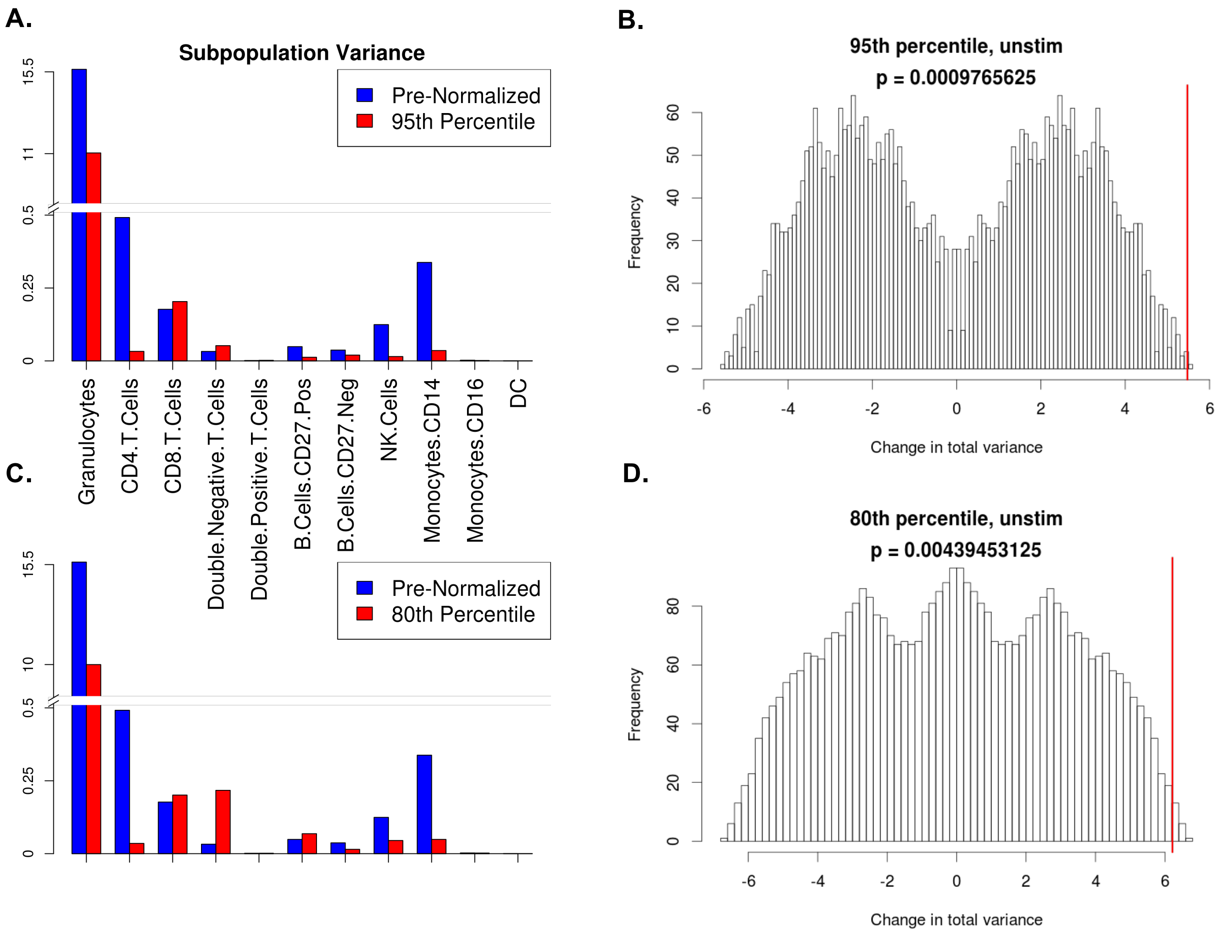

Figure S4. Manual gating subpopulation variance. Variance across all unstimulated anchor replicates in the fraction of events assigned to each population pre- and post-normalization. Manual gates were drawn based on the reference anchor (barcode 1) and applied to all other anchor replicates. **A and C.** Variance per subpopulation pre- (blue) and post-normalization (red) for 95th (**A**) and 80th (**C**) percentile normalization. **B and D.** Null distribution for change in total variance for the 95th (**B**) and 80th (**D**) percentile normalization. Red bar indicates observed change in total variance. p-value is the fraction of permutations with a change in total variance as or more extreme than that observed for unpermuted data.

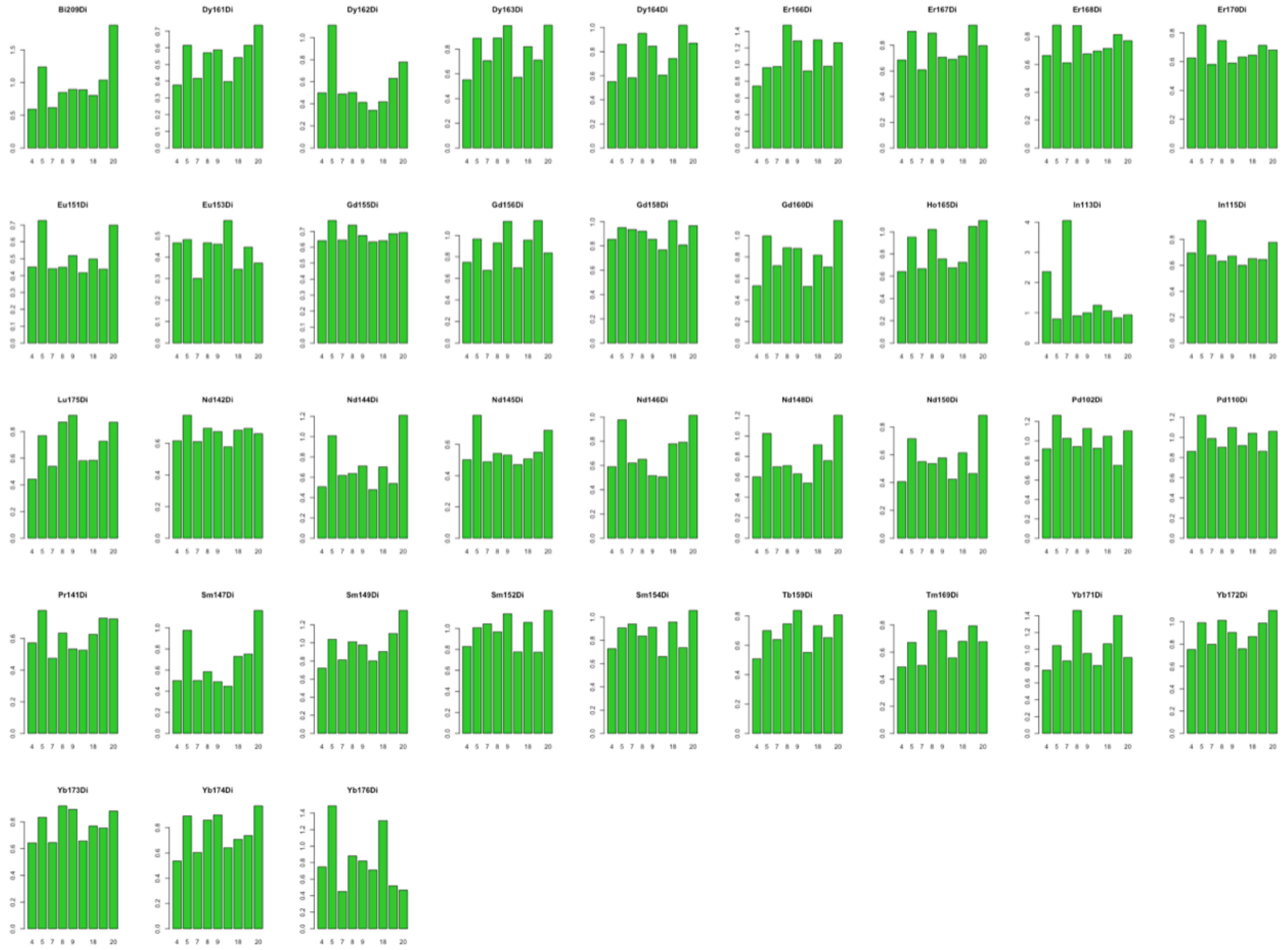

Figure S5. Scaling factors per channel per barcode set. Batch normalization was performed on a subset of 21 healthy control reference samples generated from a vaccination study conducted at Stanford University (publicly available from the flow repository http://flowrepository.org/id/FR-FCM-Z2YR). Each bar graph panel represents the relative size of the scaling factors used for each batch within each data channel. The Y-axis demonstrates the scaling factor scale, and the X-axis indicates each barcode set.

CD57_113

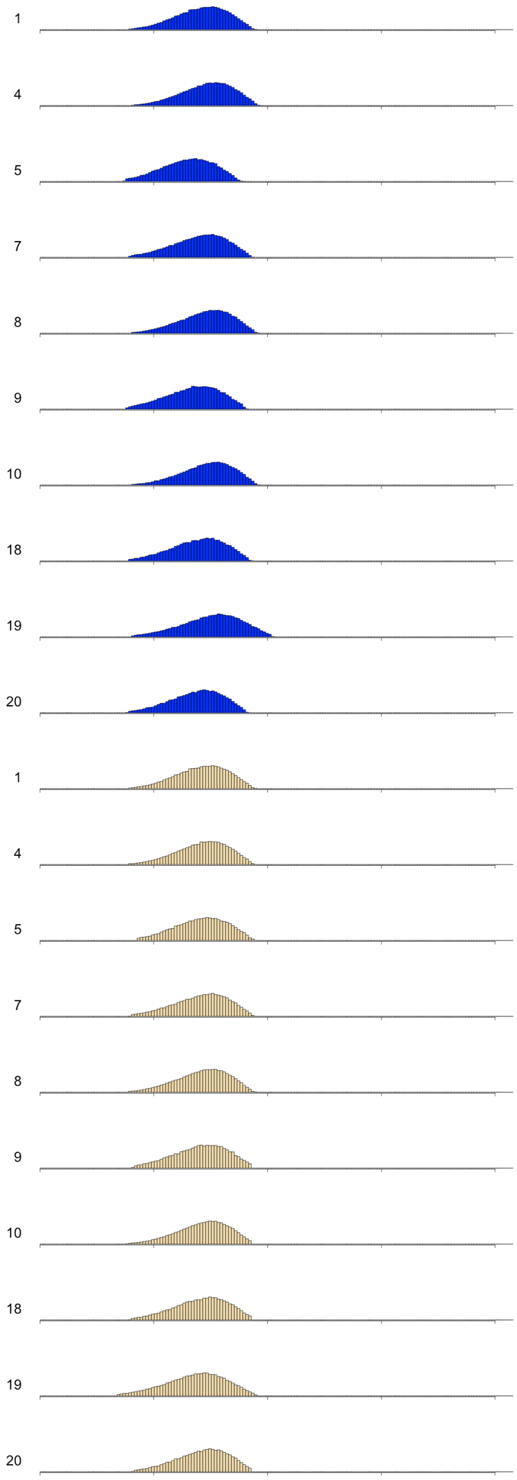

HLADR_115

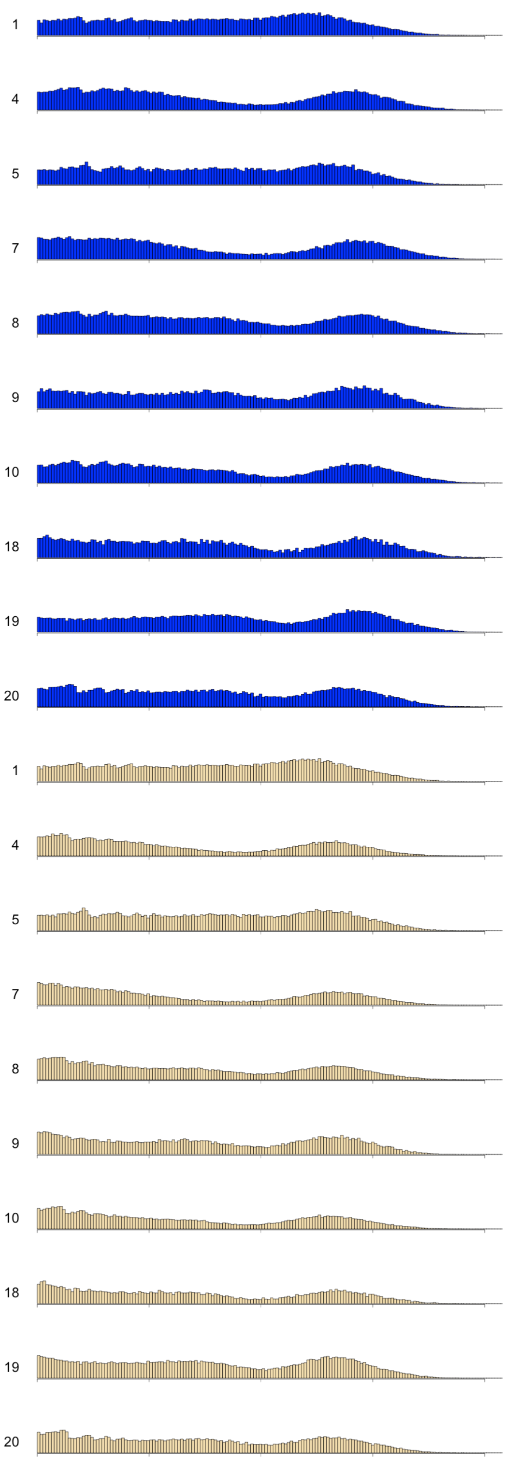

CCR6_141

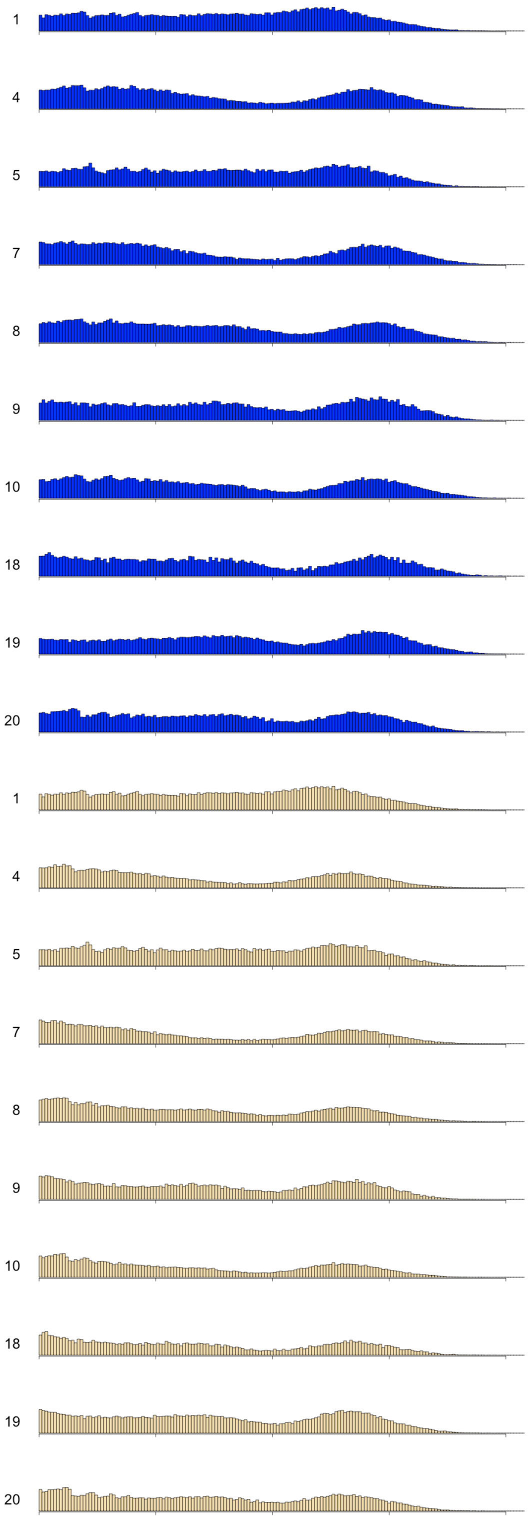

CD19_142

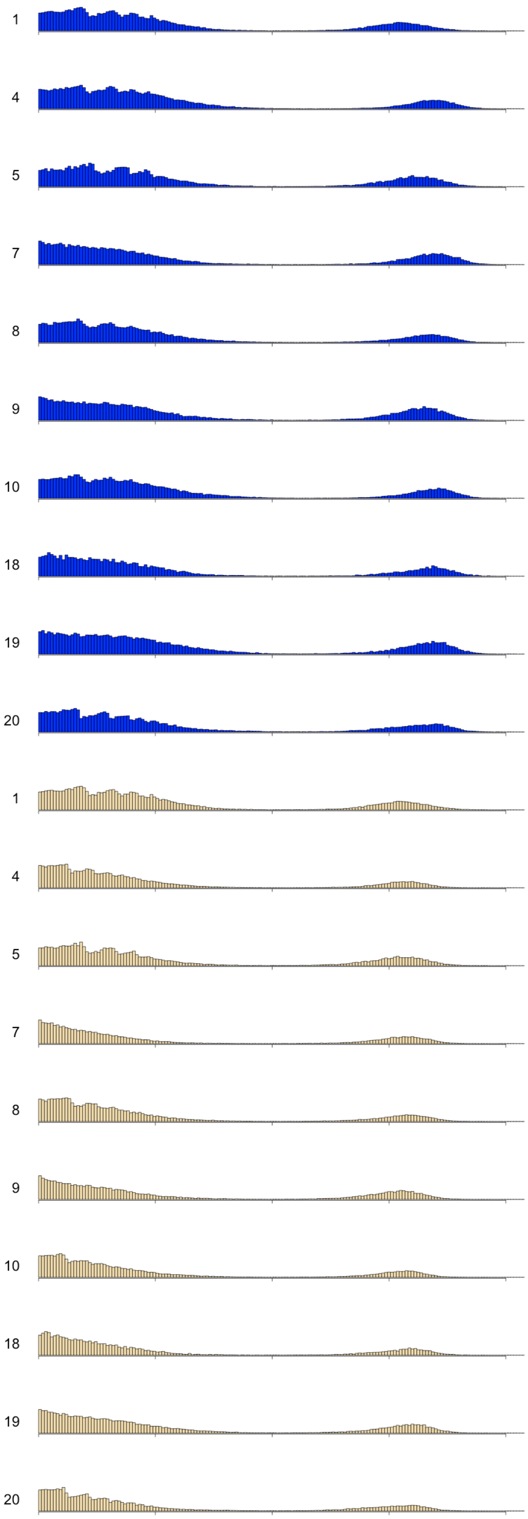

IgG_144 CD4_145

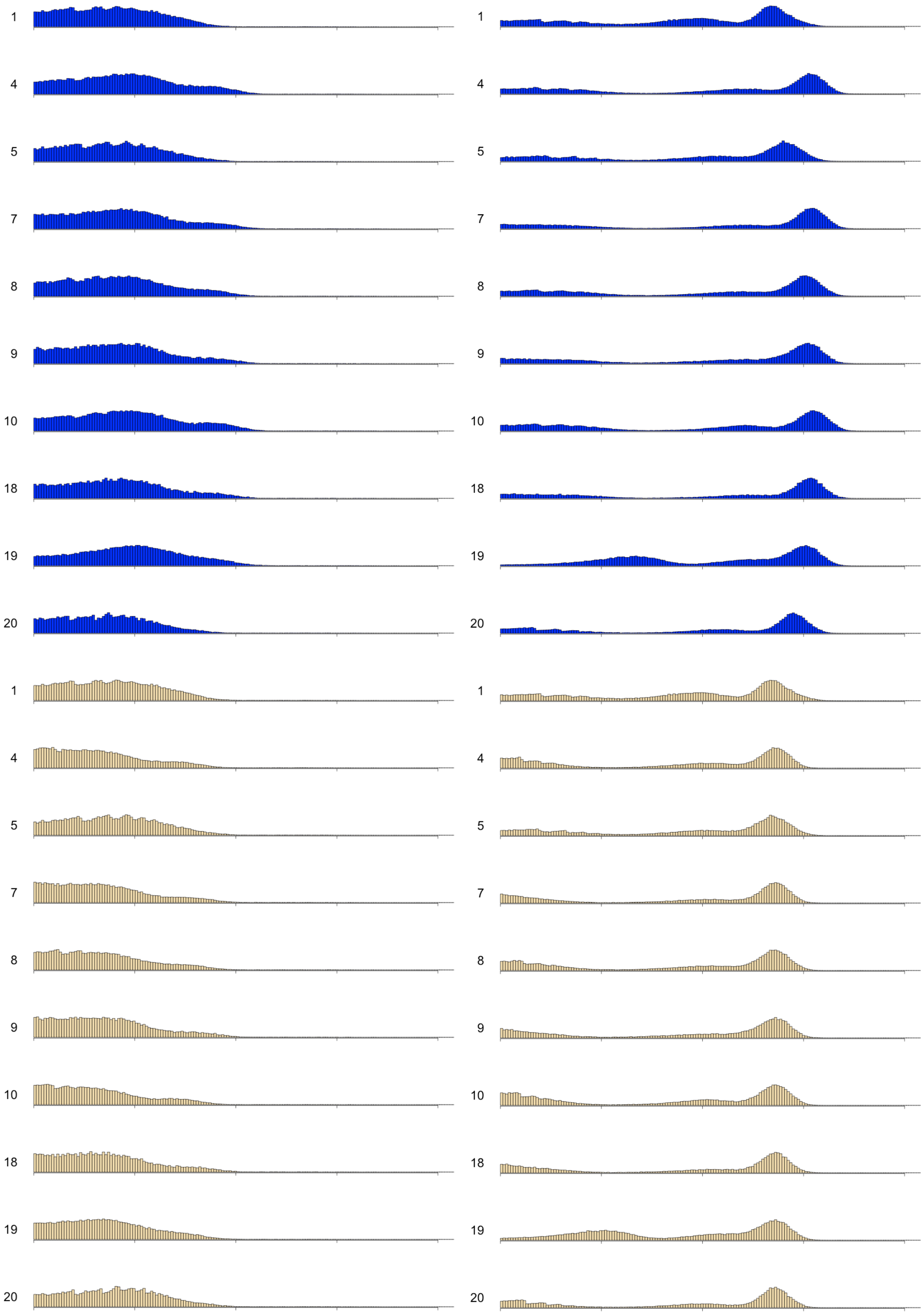

IgD_146

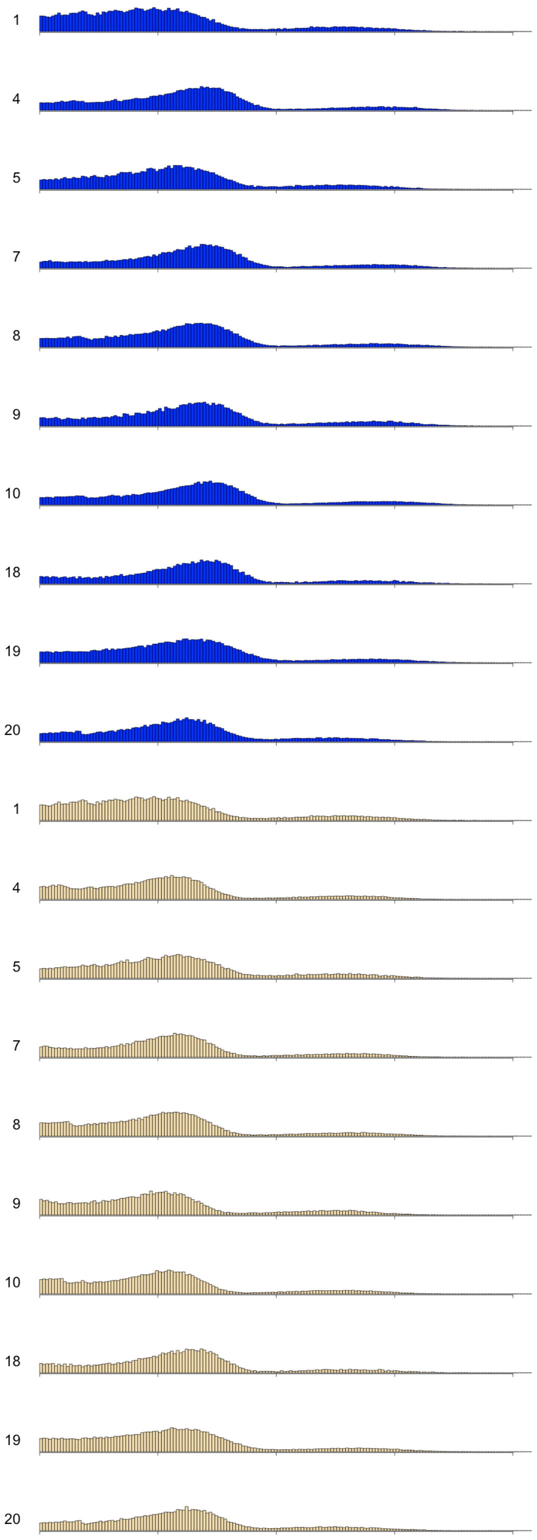

CD20_147

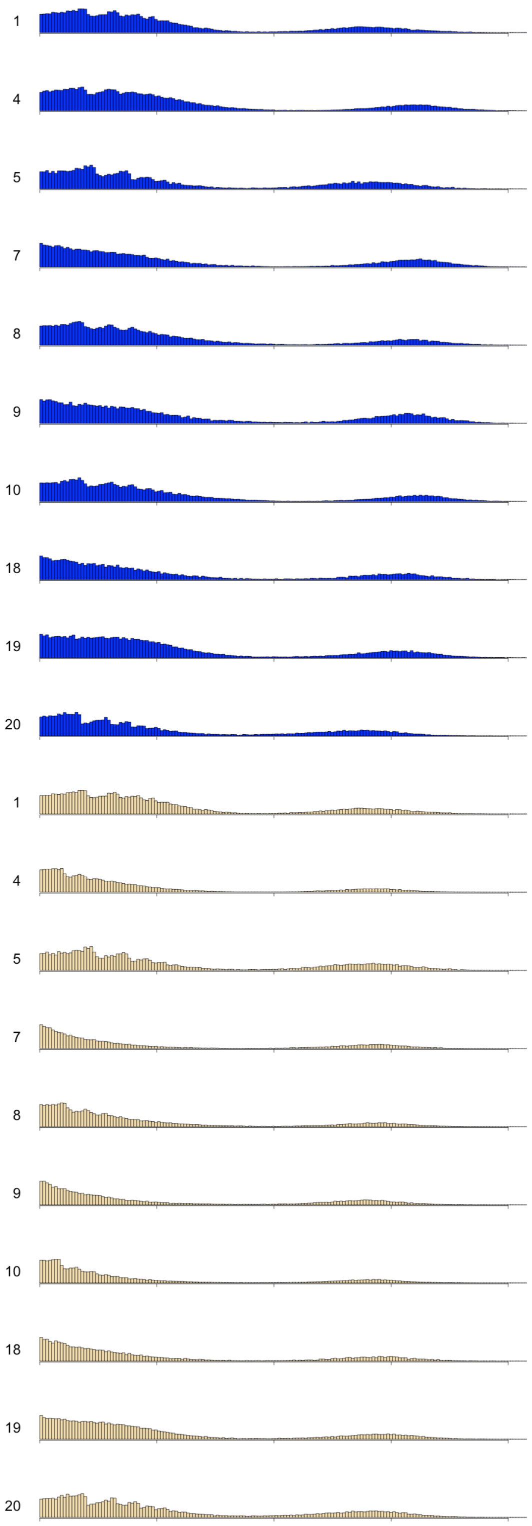

IgA_148

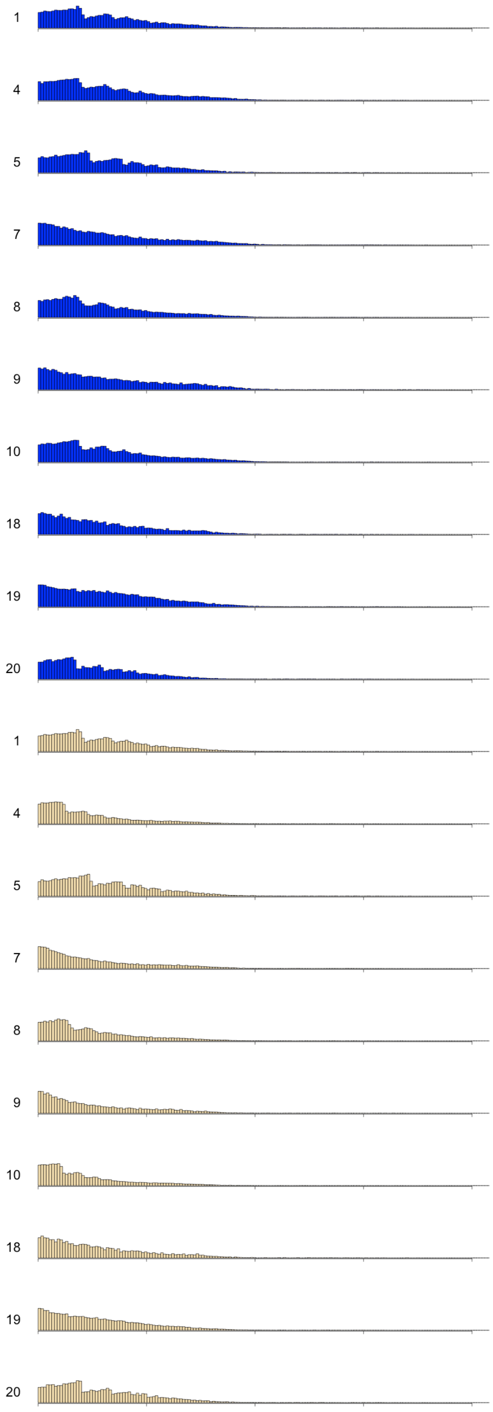

CD56_149

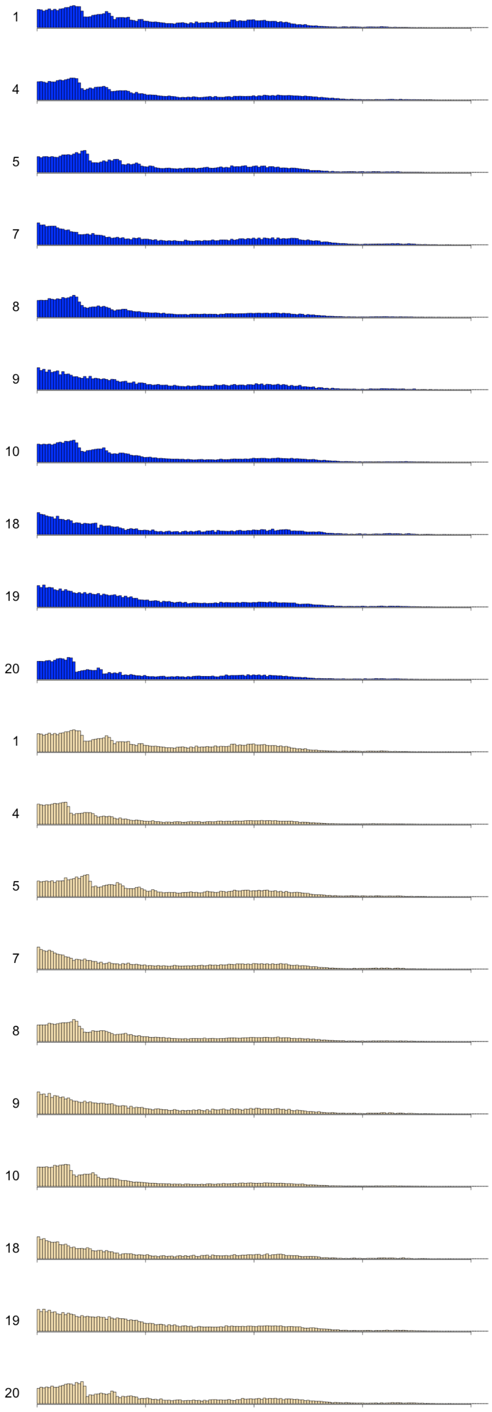

CD86_150

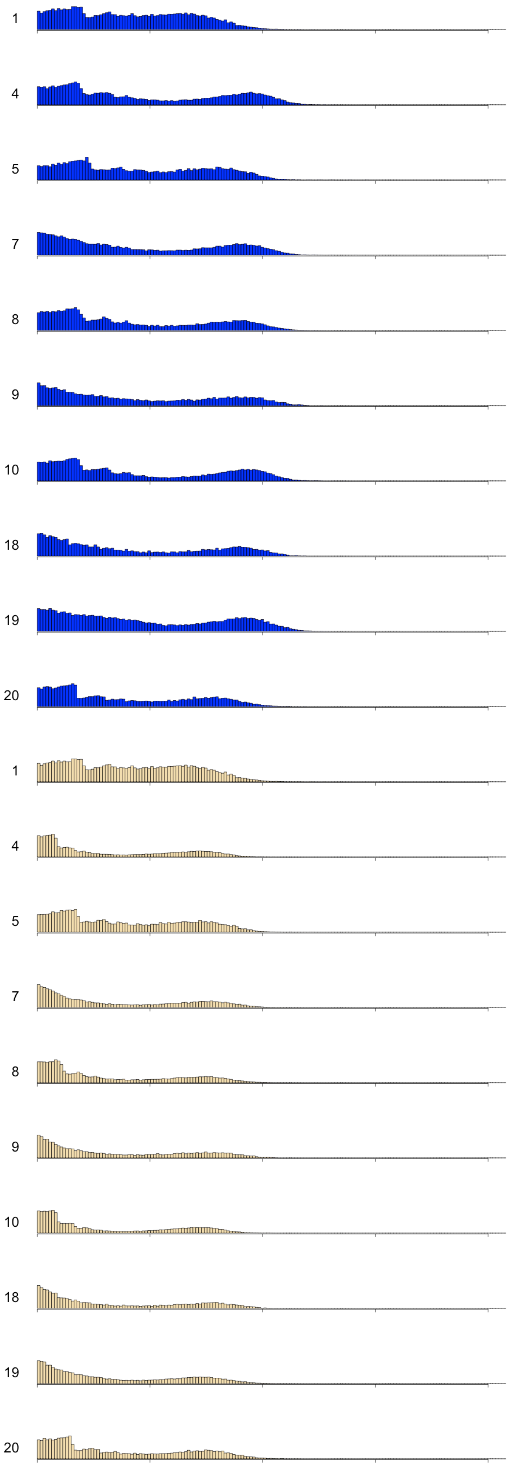

ICOS_151

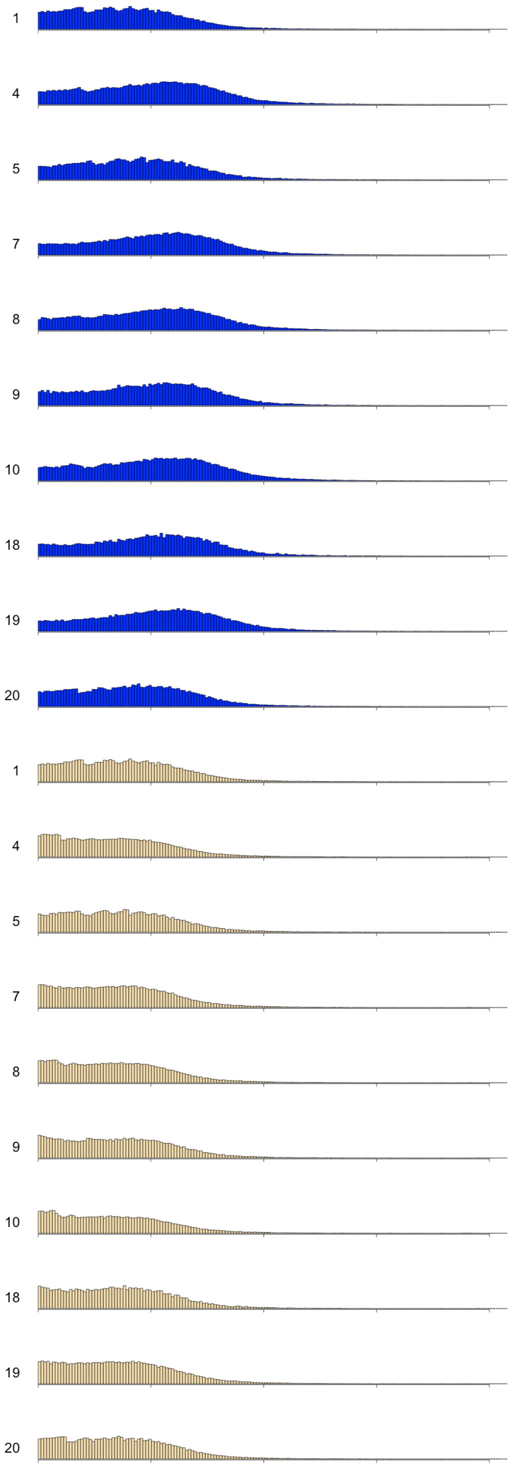

TCRgd_152

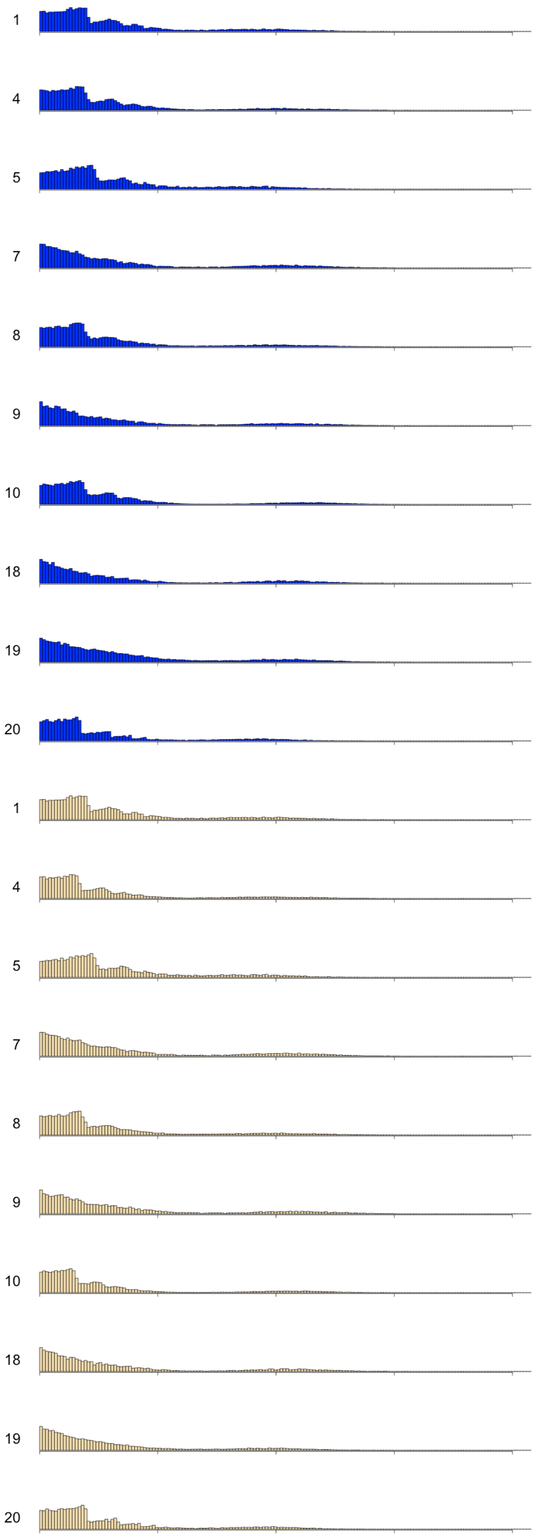

CD45RA_153

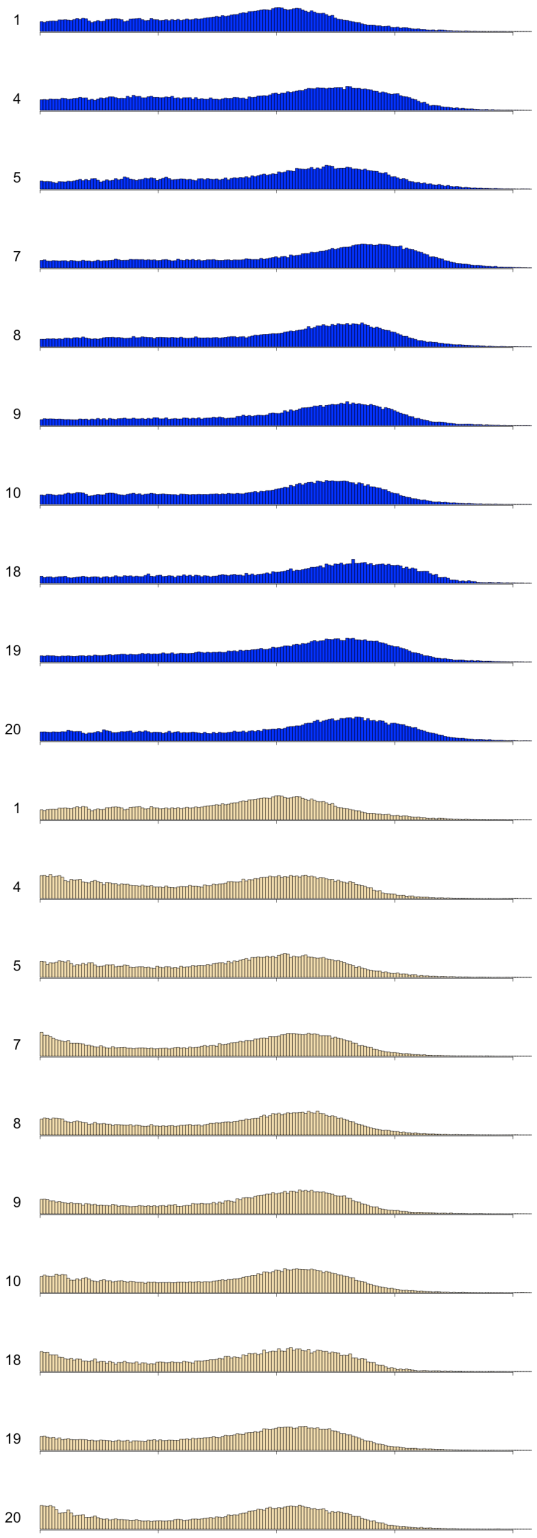

CD123_154

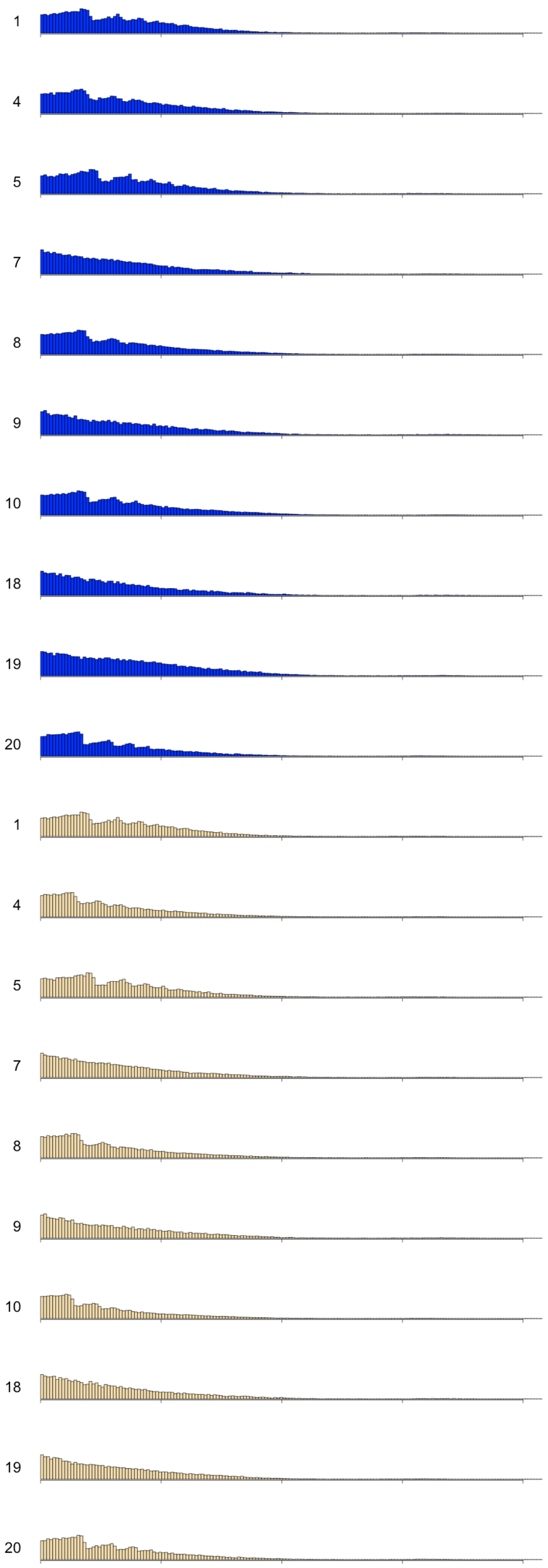

CD27_155

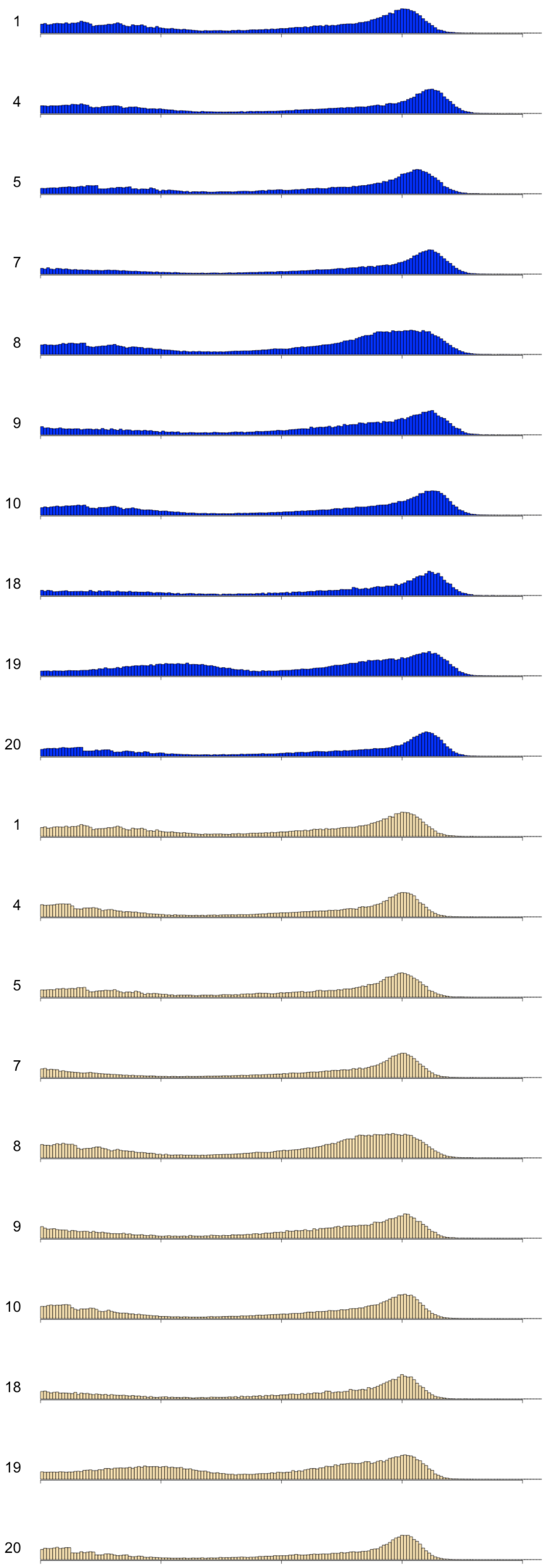

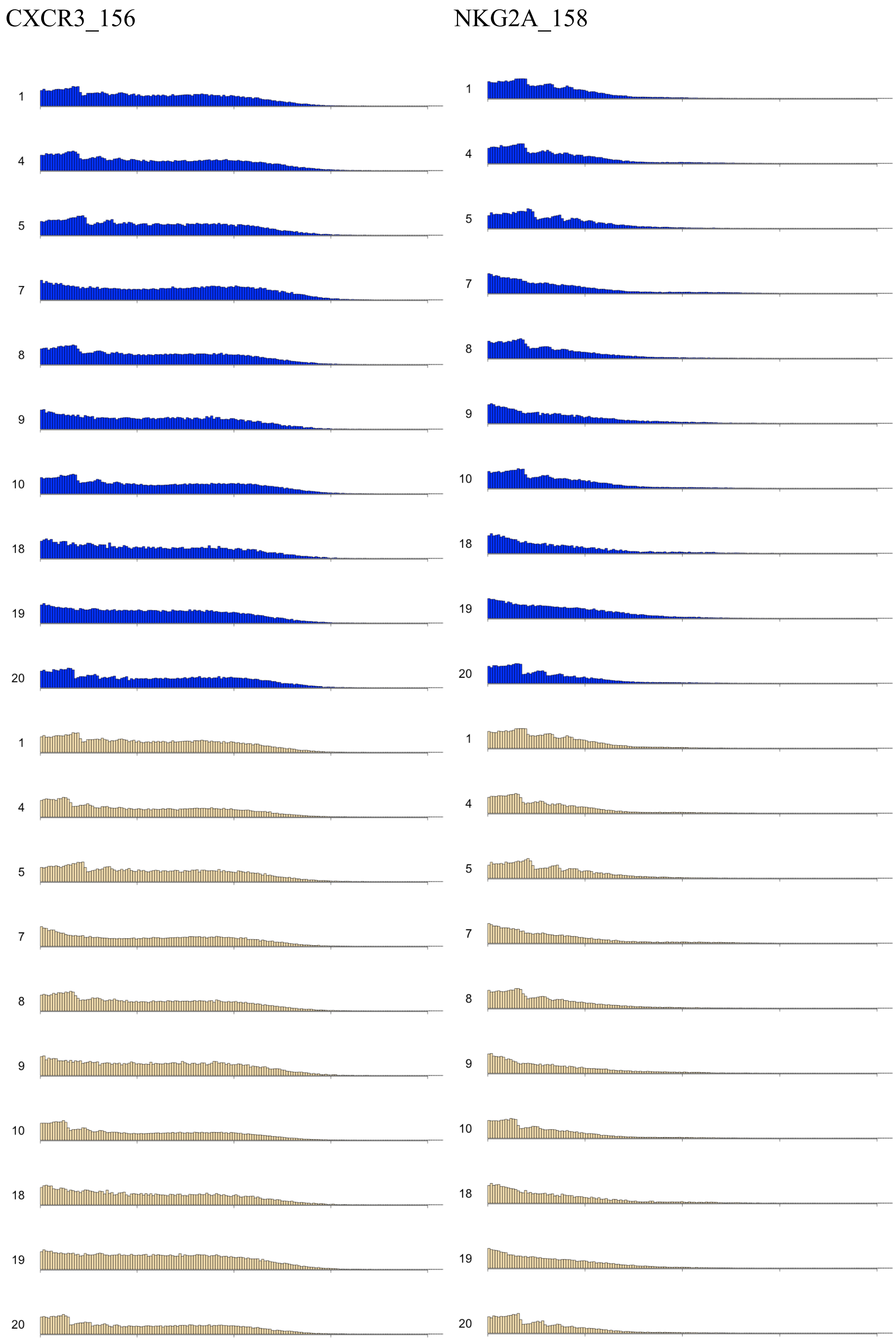
CXCR3_156
NKG2A_158
1
4
5
7
8
9
10
18
19
20
1
4
5
7
8
9
10
18
19
20

CD11c_159

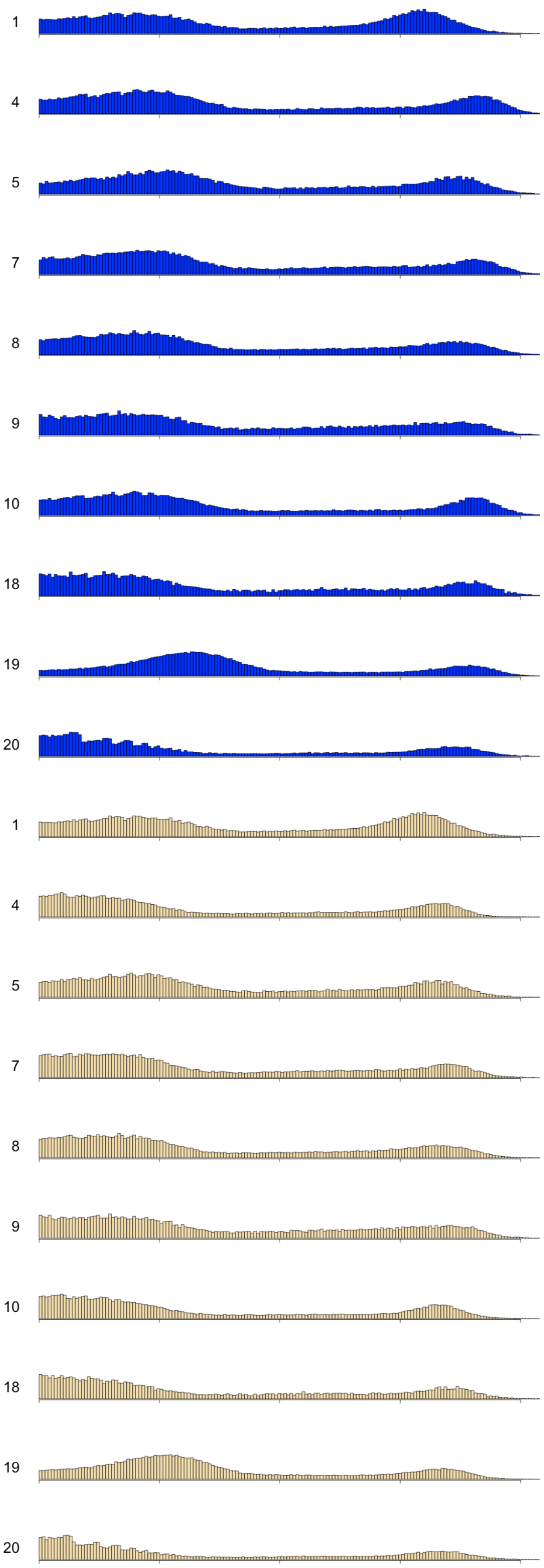

CD14_160

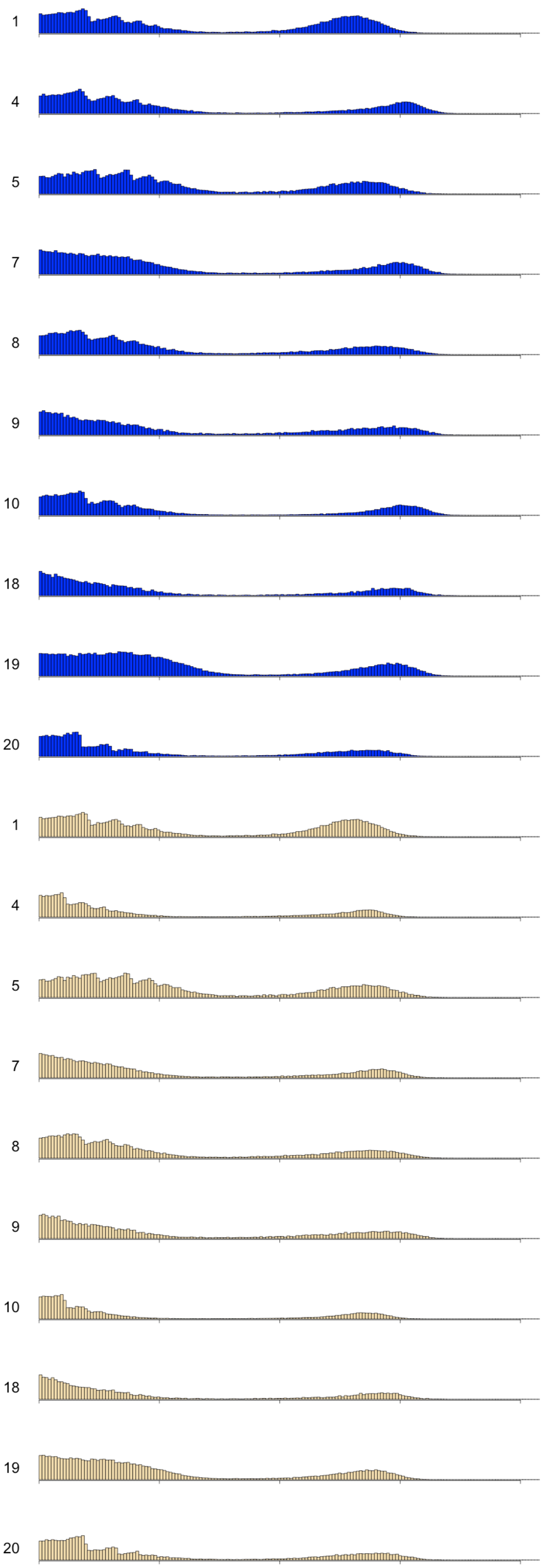

CD26_161

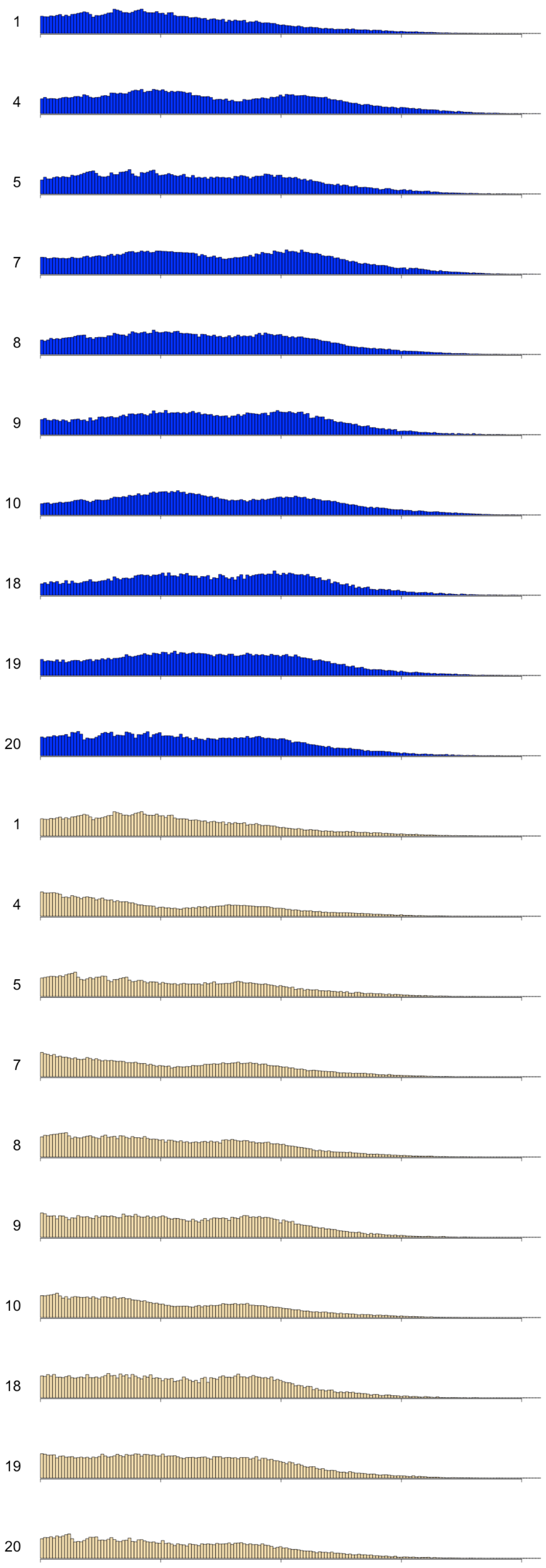

CD8a_162

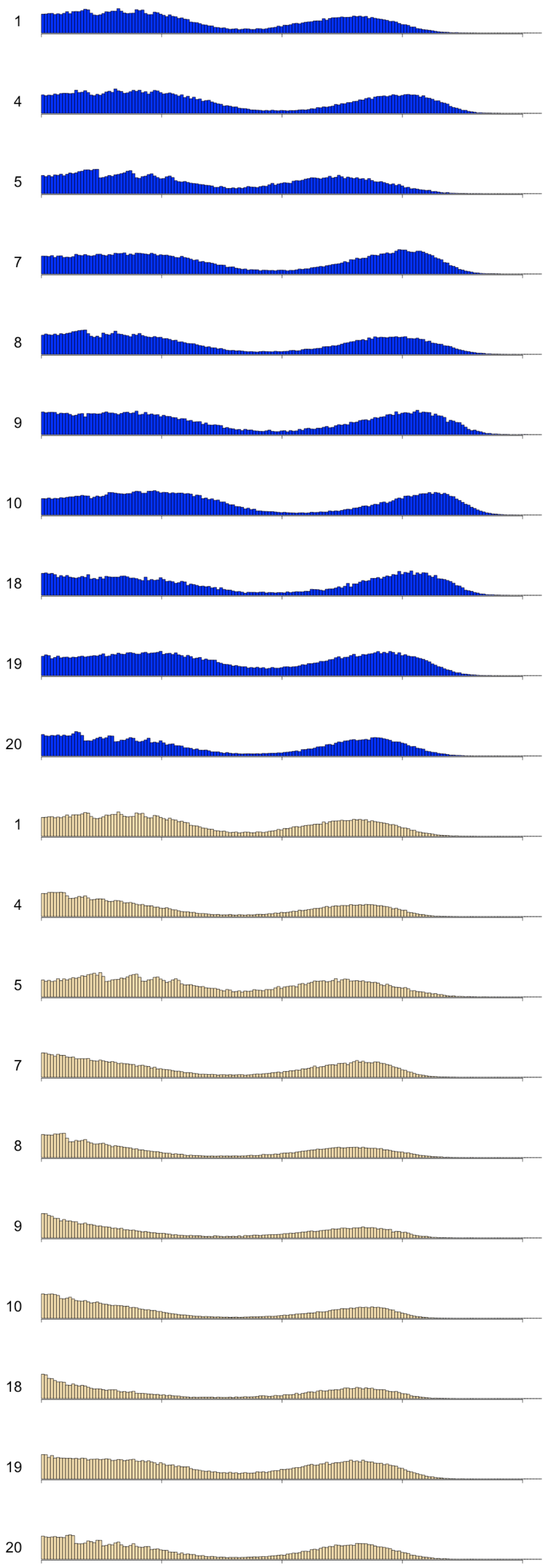

CD33_163

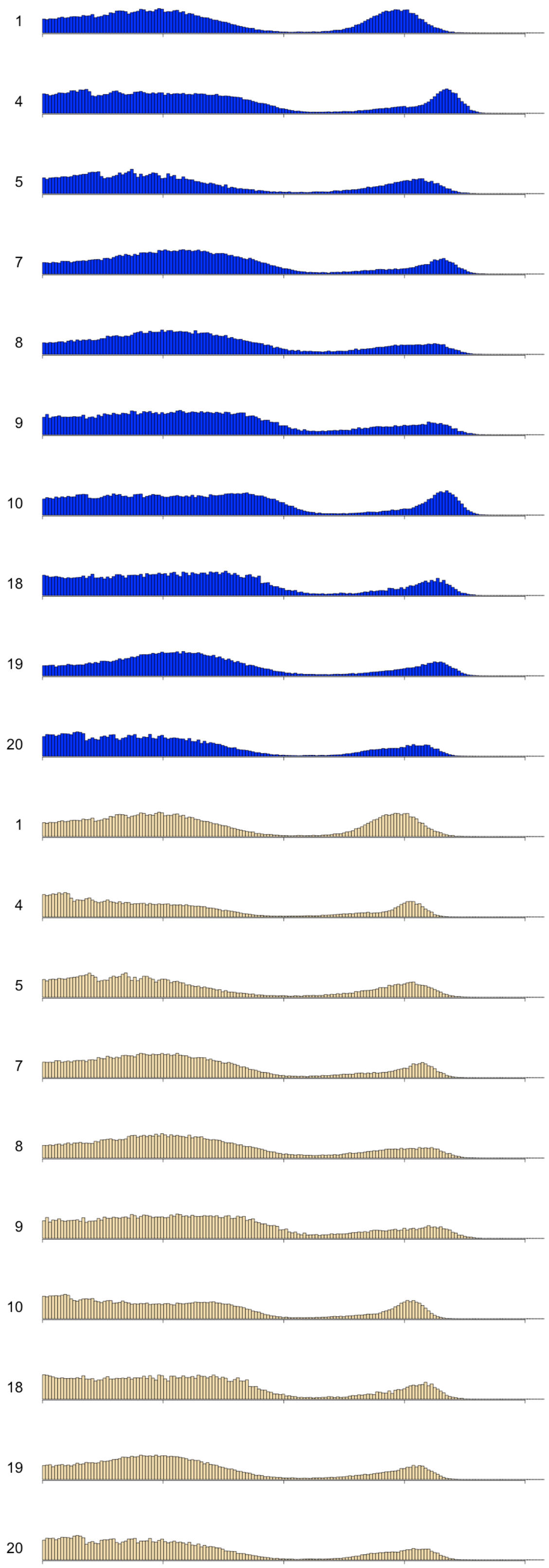

CD161_164

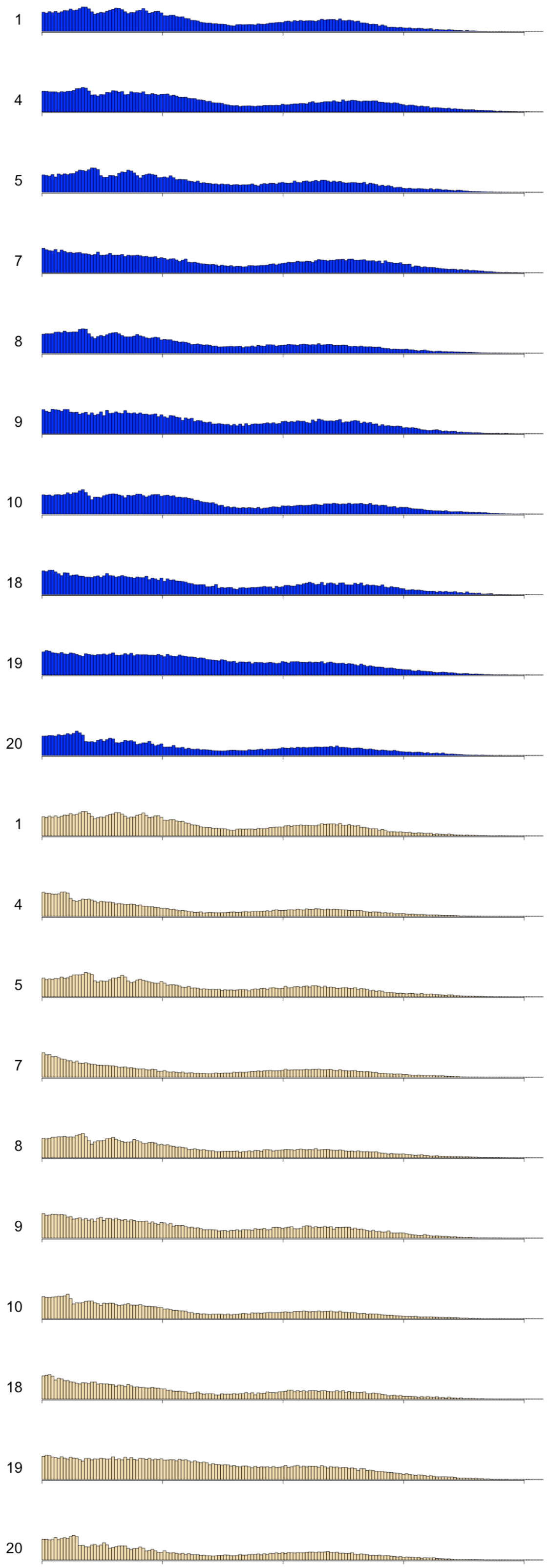

CD127_165

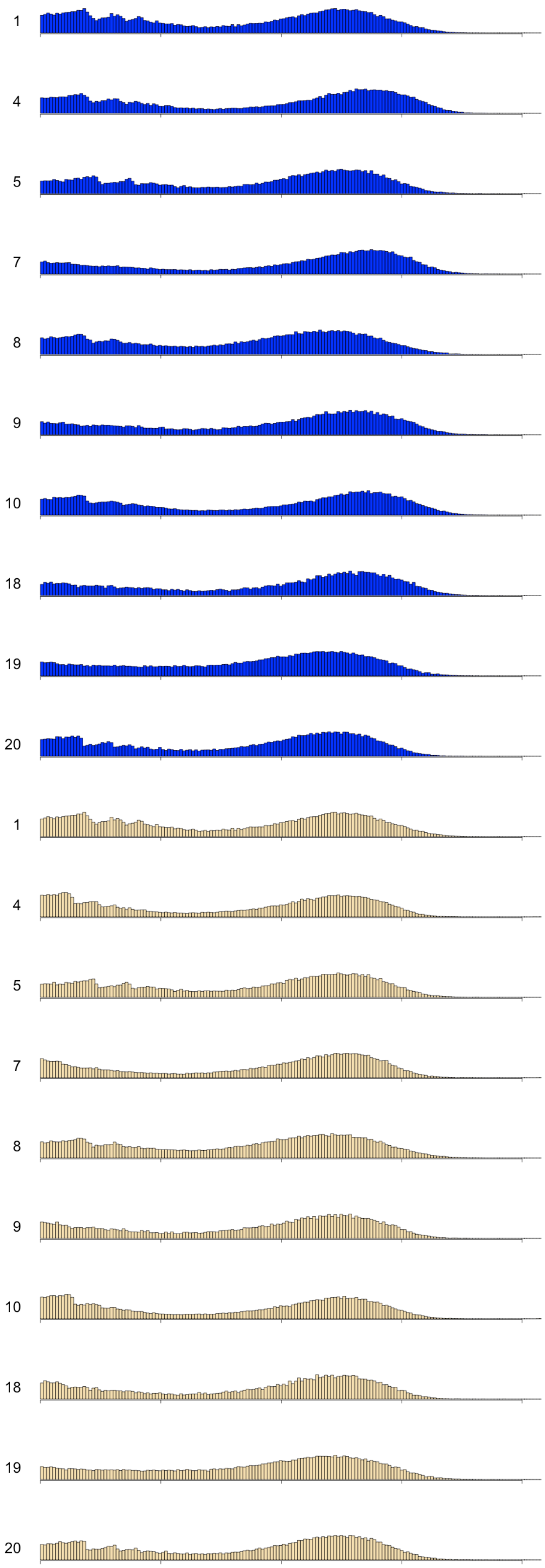

CCR10_166

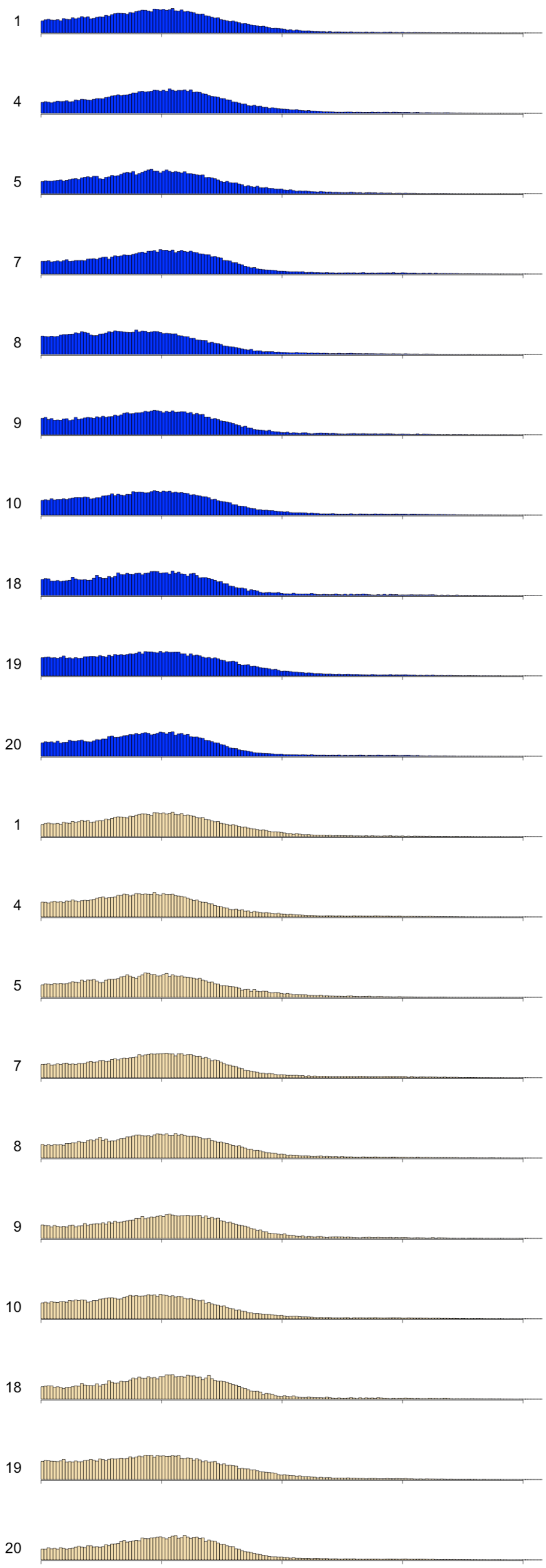

CCR7_167

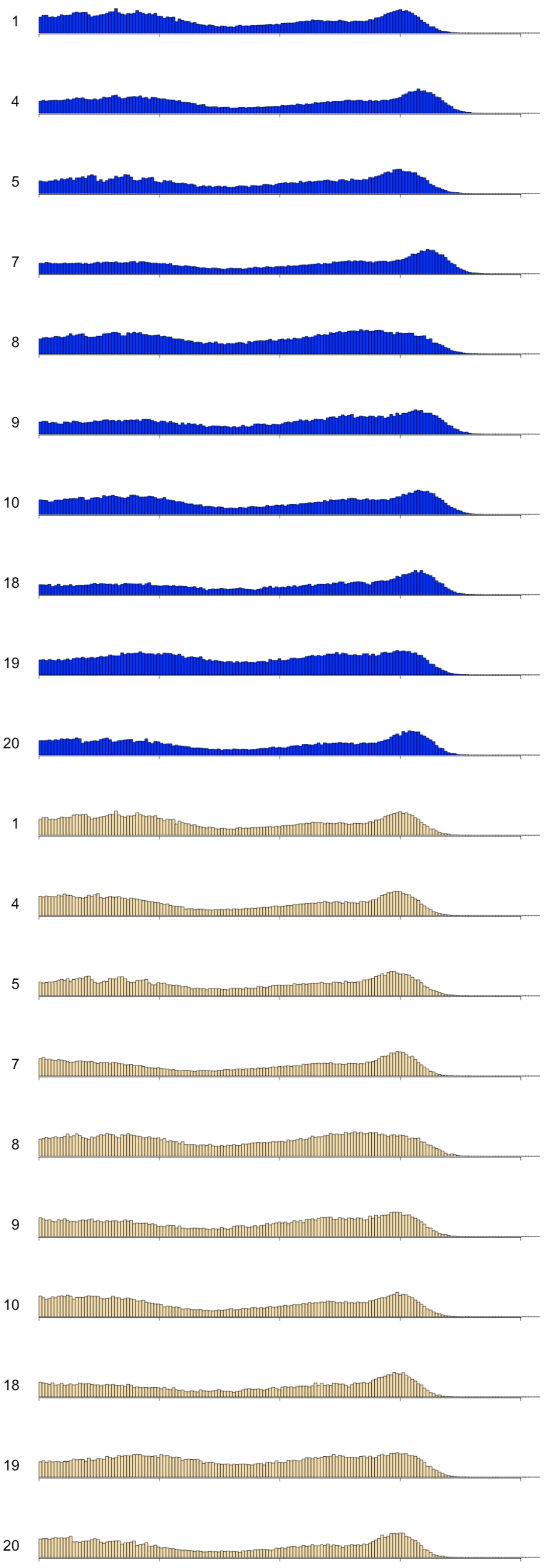

CCR9_168

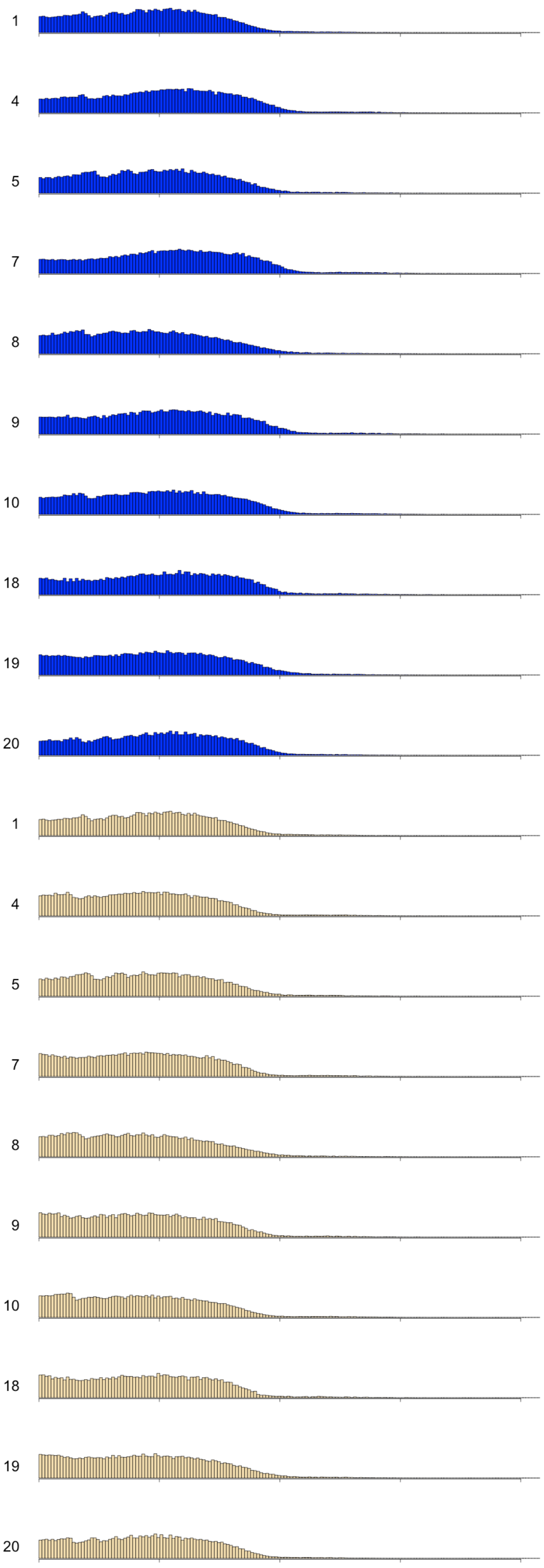

CD25_169 CD3_170

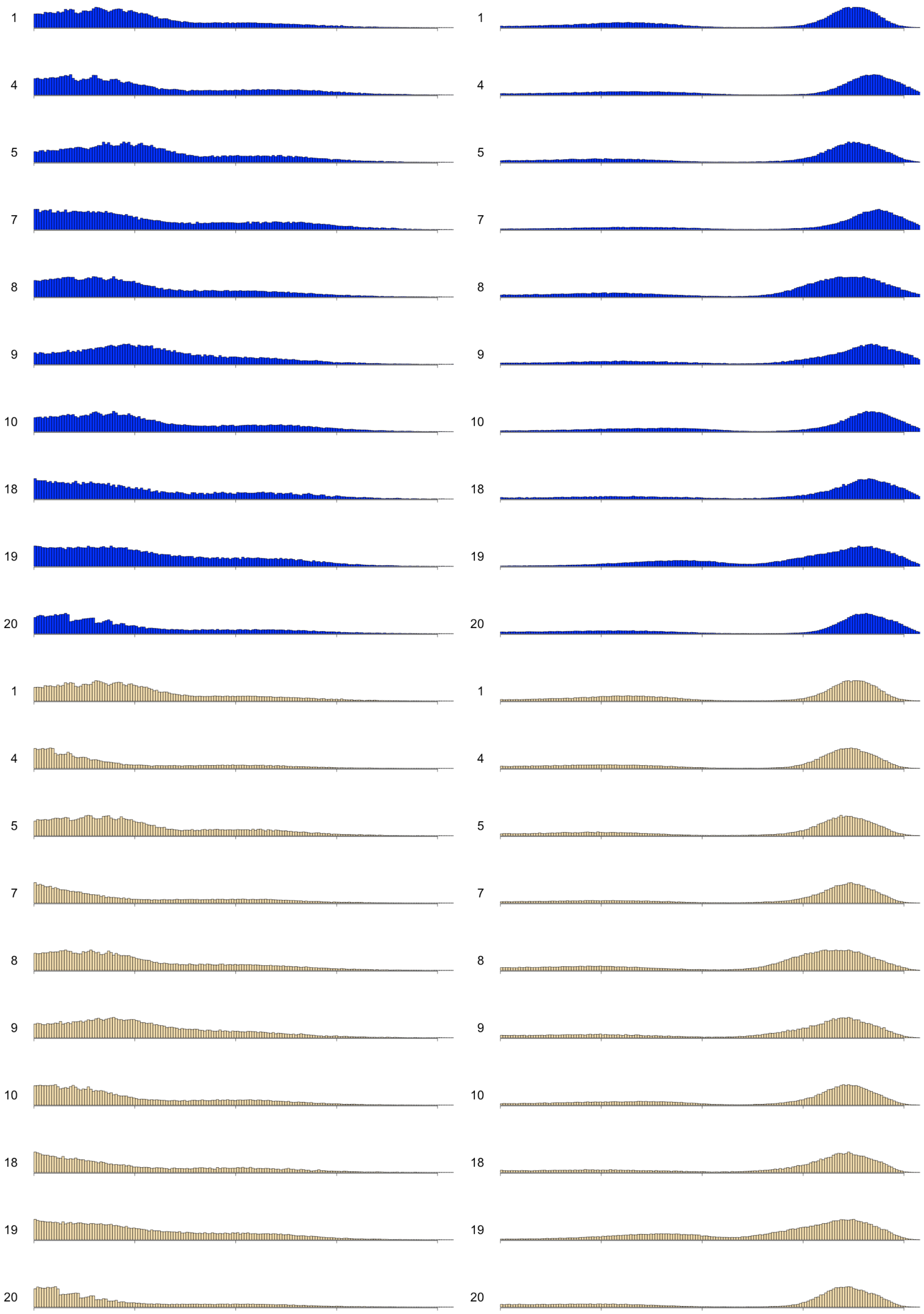

CXCR5_171

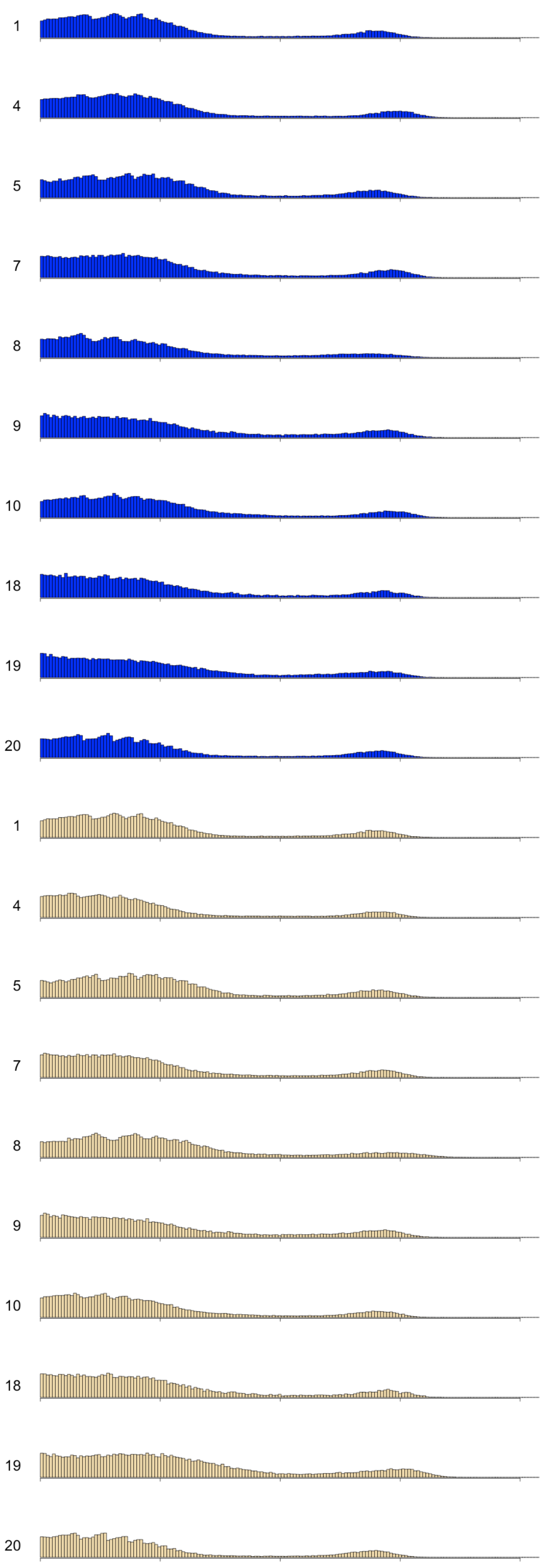

CD38_172

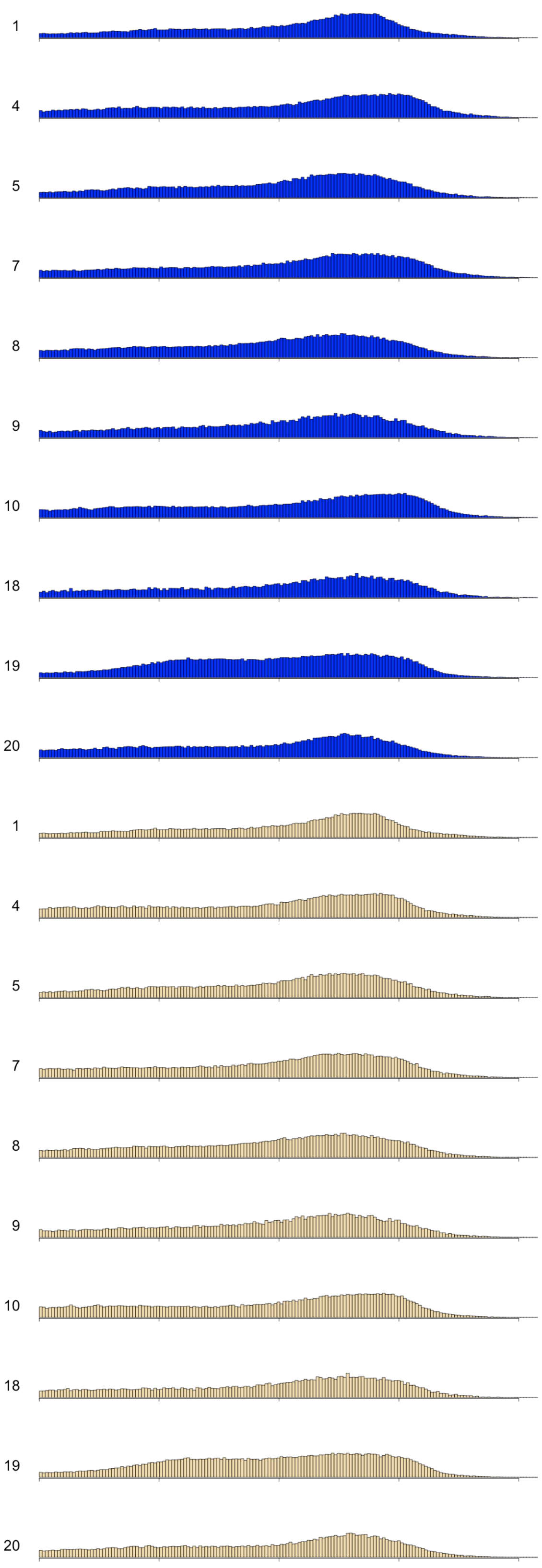

A4b7_173

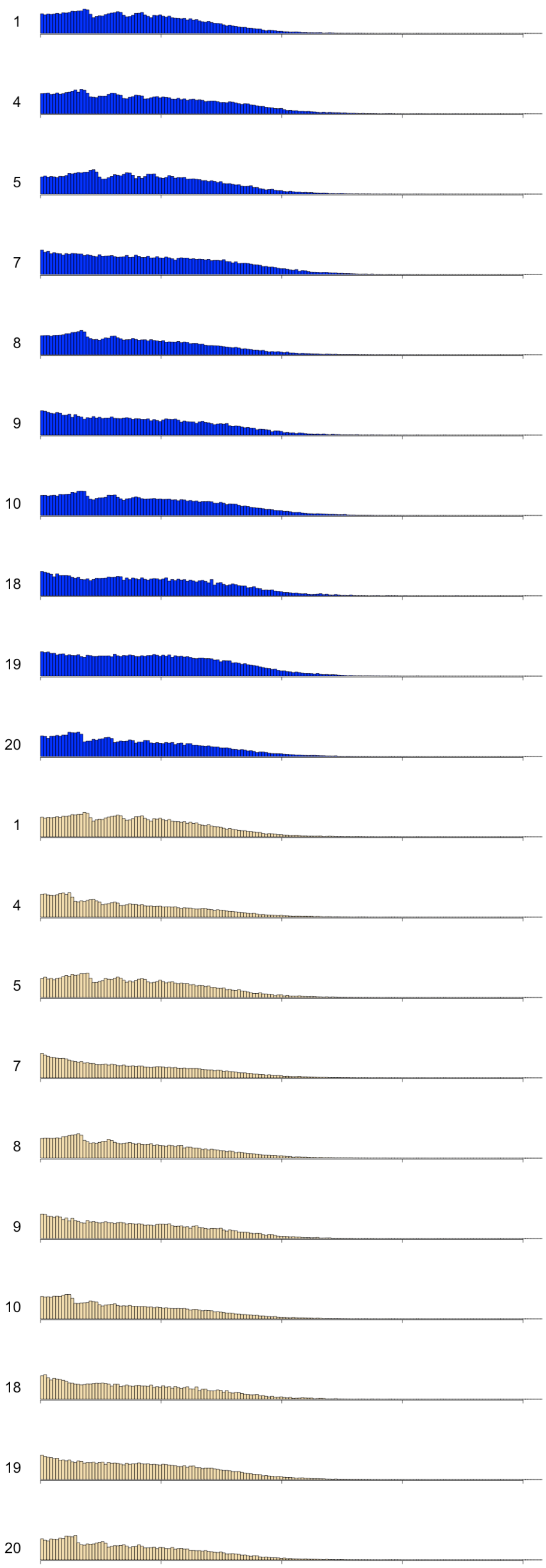

PD-1_174

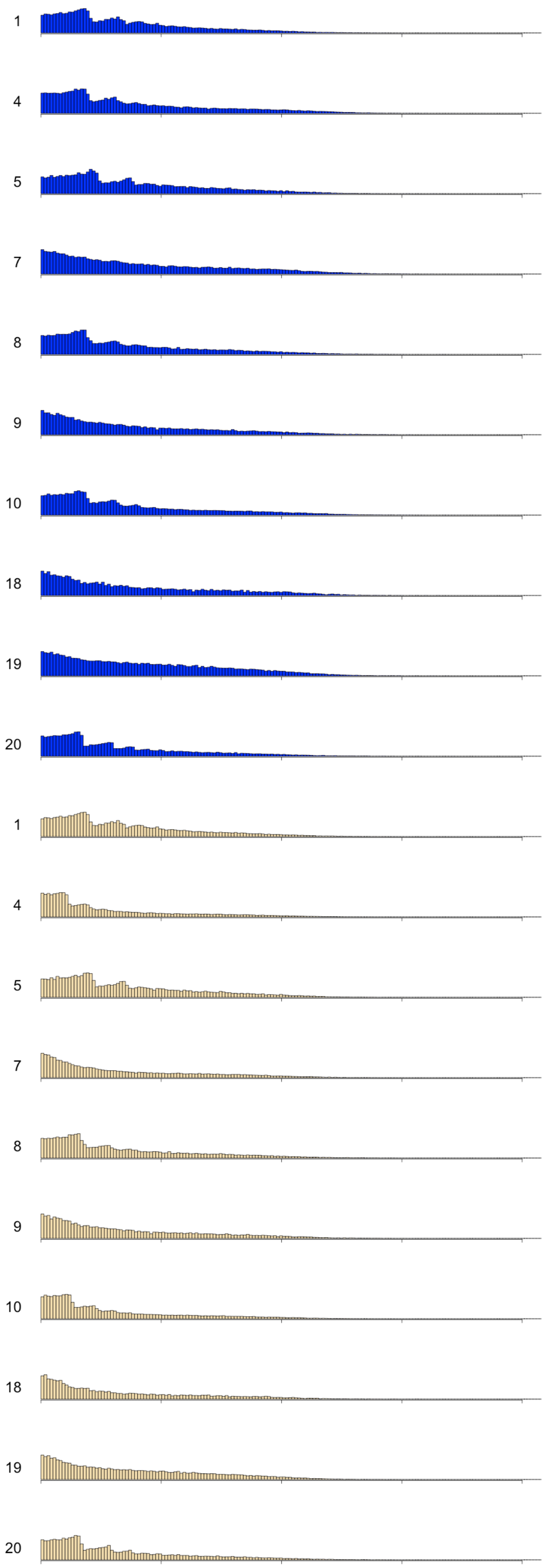

CD62L_175

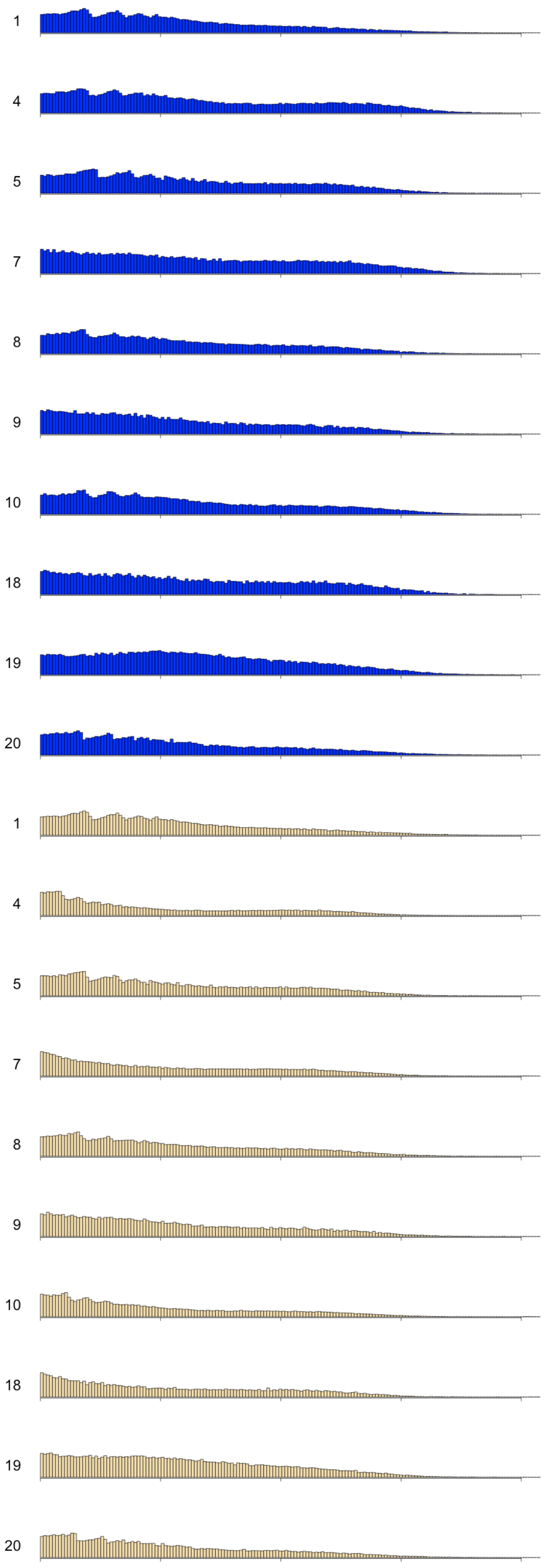

CLA_176

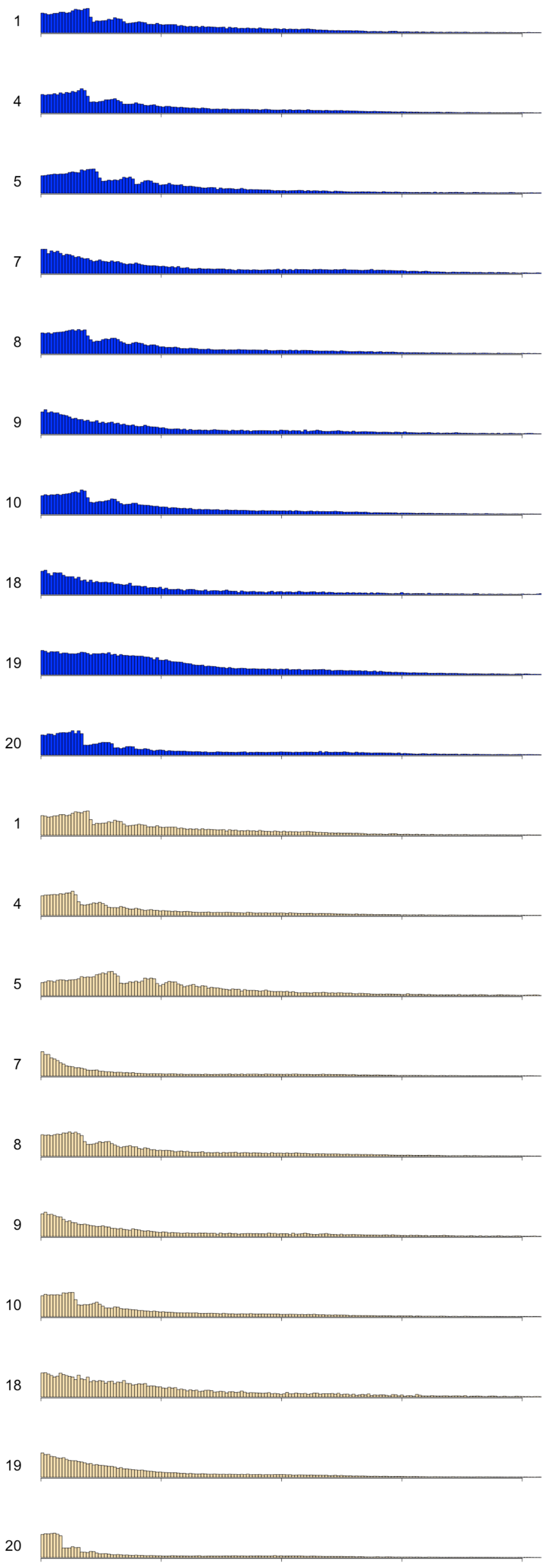

Figure S6. Signal intensity distribution plots. Batch normalization was applied to the publicly available dataset as described in Figure S5. Each panel demonstrates the signal intensity distribution for that channel pre- (blue) and post- (gold) normalization. The numbers on the left denote the specific barcode set.

Supplementary Tables

Parent	Child	Marker 1	Marker 2
single cells 2	Granulocytes	CD66+	
single cells 2	Lymphocytes	CD66-	CD45+
Lymphocytes	T cells	CD45+	CD3+
T cells	CD4+ T cells	CD4+	CD8-
T cells	CD8+ T cells	CD4-	CD8+
Lymphocytes	Non T cells	CD45+	CD3-
Non T cells	Non B cells	CD19-	
Non T cells	B cells	CD19+	HLADR+
Non B cells	CD45RA+ CD7+ NK cells	CD45RA+	CD7+
Non B cells	CD45RA- CD7-	CD45RA-	CD7-
CD45RA- CD7-	CD11c+ monocytes	CD11c+	CD11b-
CD45RA- CD7-	HLADR+ CD11c- Monocytes	CD11c-	HLADR+
HLADR+CD11c- Monocytes	pDCs	CD123+	HLADR+
CD11c+ Myeloid	CD14 Monocytes	CD14+	CD16-
CD11c+ Myeloid	CD16 Monocytes	CD16+	CD14-
CD11c+ Myeloid	CD14- CD16-	CD14-	CD16-
CD14- CD16-	mDCs	CD1c+	HLADR+
B cells	CD27hi B cells	CD27_hi	
B cells	CD27lo B cells	CD27_lo	

Table S1. Immune cell populations and their surface marker definitions. Hierarchical method for defining cell subsets using a manual gating approach. Child populations were defined from parent populations using the markers described.

	MCP1						IL1RA						MIP1B					
	Pre-Normalized		80th Percentile		95th Percentile		Pre-Normalized		80th Percentile		95th Percentile		Pre-Normalized		80th Percentile		95th Percentile	
	Unst	Stim	Unst	Stim	Unst	Stim	Unst	Stim	Unst	Stim	Unst	Stim	Unst	Stim	Unst	Stim	Unst	Stim
1	11.3	904	11.1	875	11.4	891	5.08	545	4.91	512	5.09	623	2.7	390	2.68	377	2.7	403
2	6.62	503	17.3	1327	12.8	958	4.21	305	6.55	464	6.59	504	2.06	235	2.79	333	2.41	281
3	6.89	667	20.8	2089	18	1715	3.17	427	4.41	530	4.43	659	2.06	308	2.67	417	2.57	415
4	12.6	698	32.4	1901	28.5	1567	5.12	387	6.68	480	7	561	2.63	302	3.36	398	3.08	367
4	7.84	699	20.9	1885	20.6	1821	3.18	424	3.39	471	3.97	585	2.26	336	2.44	374	2.52	392
6	10.2	620	22.2	1011	18.6	878	3.48	427	4.78	482	5.23	603	2.86	361	3.01	338	2.66	312
7	4.62	300	32.9	1718	25.5	1349	2.33	184	6.66	465	6.69	532	1.6	165	3.14	355	2.76	320
8	10.2	702	24.7	1176	23.4	1172	3.81	340	5.8	399	7.11	562	2.85	335	2.99	298	3.22	347
9	4.5	515	20	1657	16.9	1535	2.23	312	3.7	557	3.9	556	2.08	270	2.54	316	2.52	326
10	11.1	917	20.6	1164	22.1	1310	3.47	359	3.91	378	4.75	499	3.45	407	2.89	302	3.21	360
11	6.99	671	19.7	1296	17.2	1200	4.85	289	6.22	335	6.33	340	2.07	334	2.37	339	2.2	319
12	4.24	463	25	1968	19.6	1560	5.58	256	7.87	348	7.47	359	1.95	251	2.72	335	2.53	319

Table S2. Mean signal intensity for MCP1, IL-1RA, and Mip1β in CD14^{hi} monocytes pre- and post-normalization. Pre and post normalized anchor files were manually gated and mean signal intensity for CD14hi monocytes for each of the above markers was calculated and exported using Flowjo (version 10). Both stimulated and unstimulated anchor files were used to show that the normalization procedure did not substantially alter expected functional changes within this population.

Sample:
02Feb18_Helios2_Plate3_Sample1_HIMCctrl.fcs
02Mar18_Helios2_Plate9_Sample1_HIMCctrl.fcs
02Mar18_Helios2_Plate9_Sample2_HIMCctrl_cct.fcs
02Mar18_Helios3_Plate9_Sample3_HIMCctrl.fcs
04Apr18_Helios2_Plate18_Sample2_HIMCctrl_cct.fcs
04Apr18_Helios2_Plate18_Sample3_HIMCctrl.fcs
06Apr18_Helios2_Plate19_Sample2_HIMCctrl_cct.fcs
06Apr18_Helios2_Plate19_Sample3_HIMCctrl.fcs
07Feb18_Helios2_Plate4_Sample1_HIMCctrl.fcs
07Feb18_Helios2_Plate4_Sample2_HIMCctrl.fcs
07Mar18_Helios2_Plate10_Sample1_HIMCctrl.fcs
07Mar18_Helios2_Plate10_Sample2_HIMCctrl.fcs
07Mar18_Helios2_Plate10_Sample3_HIMCctrl.fcs
09Mar18_Helios2_Plate11_Sample1_HIMCctrl_cct.fcs
11Apr18_Helios2_Plate20_Sample1_HIMCctrl.fcs
11Apr18_Helios2_Plate20_Sample2_HIMCctrl.fcs
11Apr18_Helios2_Plate21_Sample1_HIMCctrl.fcs
14Feb18_Helios2_Plate5_Sample2_HIMCctrl.fcs
14Feb18_Helios2_Plate5_Sample3_HIMCctrl.fcs
14Mar18_Helios2_Plate12_Sample3_HIMCctrl.fcs
16Feb18_Helios2_Plate6_Sample3_HIMCctrl_cct.fcs
16Mar18_Helios2_Plate13_Sample1_HIMCctrl.fcs
16May18_Helios2_Plate22_Sample3_HIMCctrl.fcs
21Feb18_Helios2_Plate7_Sample1_HIMCctrl.fcs
21Feb18_Helios2_Plate7_Sample2_HIMCctrl_cct.fcs
21Feb18_Helios2_Plate7_Sample3_HIMCctrl_cct.fcs
21Mar18_Helios2_Plate14_Sample3_HIMCctrl.fcs
23Feb18_Helios2_Plate8_Sample1_HIMCctrl.fcs
23Feb18_Helios2_Plate8_Sample2_HIMCctrl.fcs
23Mar18_Helios2_Plate15_Sample3_HIMCctrl.fcs
28Mar18_Helios2_Plate16_Sample3_HIMCctrl.fcs
30Mar18_Helios2_Plate17_Sample3_HIMCctrl.fcs
31Jan18_Helios2_Plate2_Sample2_HIMCctrl.fcs

Table S3. Subset of files from http://flowrepository.org/id/FR-FCM-Z2YR used for batch normalization analysis.
